# Inhibition potential of phenolic constituents from the aerial parts of *Tetrastigma hemsleyanum* against soluble epoxide hydrolase and nitric oxide synthase

**DOI:** 10.1080/14756366.2019.1584621

**Published:** 2019-03-04

**Authors:** Cai Yi Wang, Sunggun Lee, Hyun-Jae Jang, Xiang Dong Su, Heng-Shan Wang, Young Ho Kim, Seo Young Yang

**Affiliations:** a College of Pharmacy, Chungnam National University, Daejeon, Korea;; b Immunoregulatory Material Research Center, Korea Research Institute of Bioscience and Biotechnology, Jeongeup-si, Jeonbuk, Korea;; c State Key Laboratory for Chemistry and Molecular Engineering of Medicinal Resources, School of Chemistry and Pharmaceutical Sciences, Guangxi Normal University, Guilin, People's Republic of China

**Keywords:** *Tetrastigma hemsleyanum*, vitaceae, phenolics, soluble epoxide hydrolase, nitric oxide synthase

## Abstract

The aerial parts of *Tetrastigma hemsleyanum* (APTH) have been used as a functional tea in China. The purpose of the current study was to identify the bioactive constituents with inhibitory activity against soluble epoxide hydrolase (sEH) and inducible nitric oxide synthase (iNOS), which are jointly considered potential therapeutic targets for vascular system diseases. In the present study, 39 compounds (**1**–**39**) were isolated from the APTH. Among them, compounds **8**, **10**, **12**, **16**, **17**, **19**, and **32** displayed potential activities, with IC_50_ values ranging from 4.5 to 9.5 µM, respectively, and all in non-competitive inhibition mode. Compounds **5**, **10**, **12**, **19**, and **32** displayed potent iNOS inhibitory effects, with IC_50_ values ranging from 15.6 to 47.3 µM. The results obtained in this work contribute to a better understanding of the pharmacological activities of *T*. *hemsleyanum* and its potential application as a functional food.

## Introduction

Soluble epoxide hydrolase (sEH) is widely distributed in mammalian tissues and exerts potent biological activities on the cardiovascular and urinary systems^1^. sEH is responsible for the hydrolysis of epoxyeicosatrienoic acids (EETs), which are endothelium-derived hyperpolarizing factors that act as regulators of vascular function^2^. sEH converts EETs to their corresponding diols (dihydroxyeicosatrienoic acids) and reduces their effects on the cardiovascular system via vasodilation, antimigration of vascular smooth muscle cells, and anti-inflammatory actions. Thus, sEH has been considered a potential therapeutic target for vascular diseases^3^.

Endothelial nitric oxide (NO), a key mediator in the pathology of vascular and inflammatory diseases, is synthesised by inducible NO synthase (iNOS)^4^ ^,^ ^5^. NOS activity can be regulated by a kinase- and phosphatase-dependent pathway, which produces NO that is required for physiological processes^6^. Dysregulation of NOS can lead to either apoptotic or necrotic cell death, which has been implicated in the pathogenesis of atherosclerosis, hypertension, rheumatoid arthritis, and multiple sclerosis^7^. It was recently determined that the phosphatase domain of sEH plays an important role in the regulation of NOS activation and sEH may play a potent role in the regulation of NOS activity via abrogating the sEH–NOS interaction by a specific c-Src kinase^8^. Thus, the search for multi-target inhibitors of the nodes in a complex network of kinase-dependent pathways incorporating both sEH and NOS has become an effective approach for developing innovative therapies for diseases involved in the multistage process of carcinogenesis^9^.

*Tetrastigma hemsleyanum* Diels et. Gilg belongs to the grape family Vitaceae and is an herbaceous perennial species native to China^10^. It is a well-known edible plant distributed widely in China known as “Sanyeqing,” which is commonly used in folk medicine for the treatment of high fever, infantile febrile convulsion, pneumonia, snake bite, and jaundice^11^. Previous studies have examined its anticancer^12^, liver protection, antioxidant^13^, anti-inflammatory, analgesic, and antipyretic activities^14^. In addition, several studies have investigated the chemical components and biological activities of *T*. *hemsleyanum* leaves^15^ and roots^16^. Some studies have indicated that phenolic compounds isolated from the root of *T*. *hemsleyanum* inhibit a human cancer cell line^12^ and the ethyl acetate fraction (EAF) exhibits various biological activities^17^. Although *T*. *hemsleyanum* has long been used as a traditional Chinese medicine, little is known about its chemical composition^13^ ^,^ ^18^.

In the course of our continuous research on the bioactive compound screening of important edible and medicinal plants in the Karst Mountains located in Southwest China^19^ ^,^ ^20^, we performed a phytochemical study on the aerial parts of *T*. *hemsleyanum* (APTH) on both sEH and NOS inhibition. We report the isolation and structure identification of 37 constituents from the APTH and their inhibitory effects on sEH and NOS. Our work highlights the group of natural compounds in the APTH that is responsible for its cardiovascular effects. Therefore, this research will help clarify the potential contribution of these compounds to the pharmacological activities of *T*. *hemsleyanum* and provides a significant basis for expanding the use of sustainable plant products in the food and drug industries.

**Figure 1.**
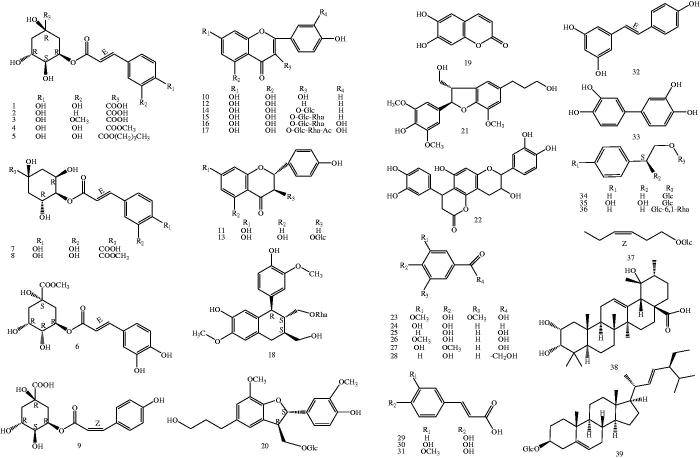
Chemical structures of isolated compounds (**1**–**39**) from *T. hemsleyanum* (Glc: glucosyl; Rha: Rhamnosyl. The configurations of all the sugar residues in the glycosides were determined as *β-*D with ^1^H NMR).

The subsequent isolation of the EtOAc-soluble fraction of the APTH resulted in the isolation of 39 known compounds, including nine chlorogenic acids (**1**–**9**), eight flavones, flavone glycosides, dihydroflavones (**10**–**17**), five phenylpropanoids (**18**–**22**), six phenolic acids (**23**–**28**), three caffeic acids (**29**–**31**), stilbene (**32**), biphenyltetrol (**33**), three phenylethanoid glycosides (**34**–**36**), hexenyl glucoside (**37**), a triterpenoid (**38**), and a steroid (**39**) (Figure 1). The isolation and structural elucidation of the compounds and the evaluation of their inhibitory effects on lipopolysaccharide (LPS)-induced NO production in macrophage RAW 264.7 cells and sEH are described.

## Material and methods

### General experimental procedures

Optical rotations were determined on a JASCO P-2000 polarimeter (Hachioji, Tokyo, Japan). The CD spectrum was recorded with a Chirascan spectropolarimeter (Applied Photophysics, U.K.). The high-resolution electrospray ionisation mass spectra (ESI-MS) were obtained from an Agilent 6530 Accurate-Mass Q-TOF LC/MS system (CA, U.S.A.). The NMR spectra were recorded on Bruker AM600 FT-NMR and Bruker BioSpin 400 NMR spectrometers (MA, USA). Column chromatography (CC) was performed on silica gel (Kiesel gel 60, 70–230 mesh and 230–400 mesh, Merck, Darmstadt, Germany), YMC*GEL (ODS-A, 12 nm S-150 µm, YMC Co., Ltd., Japan) and Sephadex LH-20 gel (Pharmacia Biotech, Sweden). TLC used pre-coated silica gel 60 F_254_ (1.05554.0001, Merck) and RP-18 F_254S_ plates (1.15685.0001, Merck, Germany). Preparative high-performance liquid chromatography (HPLC) was carried out using a Shimadzu LC-6AD (Shimadzu, Japan) instrument with a YMC-Pack ODS-A column (20 mm I.D.×250 mm, S-5 µm, 12 nm) and a SPD-20A wavelength detector at 210 nm. Soluble epoxide hydrolases (10011669) and PHOME (10009134) were purchased from Cayman (Cayman, MI, USA).

### Biological material

The aerial parts of *T. hemsleyanum* were collected from Linchuan County, Guilin City, Guangxi Zhuang Autonomous Region in July 2016. The plant was identified by Professor Shao-Qing Tang (Guangxi Normal University), and a voucher specimen (No. 20160110) was deposited at the School of Life Sciences, Guangxi Normal University in China.

### Extraction and isolation

**Figure 2.**
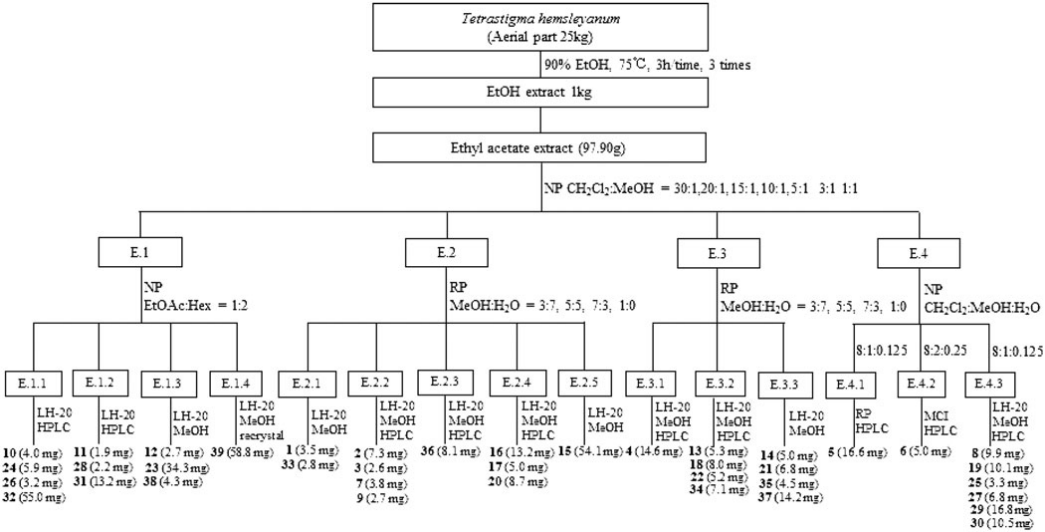
Isolation process diagram of compounds (**1**–**39**) from *T. hemsleyanum*.

The dried stems and leaves of *T. hemsleyanum* (25.0 kg) were extracted with 90% ethanol for 3 times (75 °C, 3h/time). All the filtrates were combined and concentrated to give a 1.0 kg crude extract. The crude extract was suspended in water and then respectively extracted 3 times with *n*-hexane, ethyl acetate and *n*-butanol by liquid − liquid separation. The ethyl acetate fraction (97.9 g) was further subjected to column chromatography over MCI resin, silica gel, RP-silica gel, Sephadex LH-20, and finally semipreparative RP-HPLC to obtain thirty-nine known compounds (see the process described as in Figure 2). The structures of compounds **1 **−** 39** were characterised by UV, ^1^H and ^13 ^C NMR, ESI-MS data, and comparison with published spectroscopic data. The stereochemistry of chiral compounds was determined by comparing their specific rotation, NMR or CD spectra, respectively, with that in the literature (see Supporting information S1 and S2).

### Biological study

#### sEH inhibitory activity

The enzymatic assays were performed according to methods modified from previous^21^. The 130 µL aliquot of recombinant human sEH (12.15 ng/mL) was diluted with buffer (25 mM Bis-Tris-HCl, pH 7.0 containing 0.1 mg/mL BSA) and mixed with 20 µL of ligand diluted in MeOH, and then 50 µL of 3-phenyl-cyano(6-methoxy-2-naphthalenyl) methyl ester-2-oxiraneacetic acid (PHOME, 20 µM) were added. The amount of product converted from substrate by the enzyme was measured by fluorescence photometry (excitation filter 330 nm, and emission filter 465 nm), as follows:

$$\text{Enzyme}\;\text{activity}\left(\%\right)\;=\;[S_{40}-S_{0}/C_{40}-C_{0}]\times 100$$

where, C_40_ and S_40_ are the fluorescence of control and inhibitor after 40 min, and S_0_ and C_0_ are the fluorescence of inhibitor and control at 0 min. In this testing, 12–(3-adamantan-1-yl-ureido)-dodecanoic acid (AUDA) was used as a positive control.

### Inhibition of nitric oxide production in LPS-induced RAW 264.7 cells

The inhibitory effects of compounds on lipopolysaccharide (LPS)-induced nitric oxide (NO) generation in macrophages were evaluated using literature method^22^, in which, the production of nitrite from NO oxidation was used as a measure of iNOS activity, and the nitrite present in a conditioned medium was determined using a method based on Griess reaction^23^. The viability of the microglial cells was evaluated by the MTT assay.

### Cell cultures

RAW264.7 macrophage cells purchased from the Korea Cell Line Bank (Seoul, Korea) were cultured in Dulbecco Modified Eagle’s medium (DMEM, Welgene, Daegu, Korea) supplemented with 10% FBS and 1% penicillin/streptomycin (Thermofisher Scientific, Waltham, MA, USA) under an atmosphere of 5% CO_2_ in a humidified 37 °C incubator. RAW264.7 cells seeded in 48-well plates (10^5^ cells/well) were pre-treated with the isolated compounds for 2 h and then activated with LPS (500 ng/ml) (Sigma-Aldrich Chemical Co., St. Louis, MO, USA) for 24 h. The final concentrations of test compound that cells received were 16, 31, 63, 125, 250, and 500 µM, respectively. Then supernatants were collected to determine nitrite concentration. Control cells were grown under identical conditions without addition of the test compounds or LPS.

### Cell viability

The effects of various experimental modulations on cell viability were evaluated by determining mitochondrial reductase function with the MTT assay^24^. For the determination of cell viability, 5 mg/mL of MTT was added to cells (5 × 10^4^ cells/mL in 96-well plates) for 24 h. The formazan synthesised was dissolved in DMSO and the optical density was measured at 570 nm. The optical density of the formazan formed in control (untreated) cells was considered as 100% viability.

### NO determination

Production of nitrite, a stable end product of NO oxidation, was used as an indication of NO production. The procedure for NO determination was based on the Griess reaction^25^. In a 96-well microtitration plate, an aliquot (100 µL) of each cell culture supernatant was mixed with the same volume of Griess reagent (0.1% (w/v) N-(1-naphathyl)-ethylenediamine and 1% (w/v) sulphanilamide in 5% (v/v) phosphoric acid) for 10 min at room temperature (the two parts being mixed together within 1 h of use). The absorbance of the final product was measured spectrophotometrically at 550 nm by using a Multiskan MK3 microplate reader (Thermo Fisher Scientific Inc. Waltham, MA, USA). The absorbance was referred to a nitrite standard curve to determine the nitrite concentration in supernatants.

### Statistical analysis

The enzyme inhibition parameter IC_50_ was calculated by fitting the Hill equation to the data using nonlinear regression (least squares best fit modelling) of the plot of percentage control activity versus concentration of the test inhibitor using GraphPad Prism 6.0 (GraphPad Software Inc., San Diego, CA). All data represented the mean ± SD of at least three independent experiments performed in triplicates. Statistical significance was indicated as determined by one-way ANOVA followed by Dunnett's multiple comparison test using GraphPad Prism 6 programme (GraphPad Software Inc., San Diego, CA, USA).

## Results and discussion

### Extraction, Isolation, and identification

Thirty-nine compounds were obtained from the EAF of the APTH ethanol extract by silica gel column chromatography (Sephadex LH-20) and semipreparative reverse phase high-performance liquid chromatography (Figure 2). The compounds were identified by ultraviolet, ^1^H- and ^13^C-nuclear magnetic resonance (NMR), and electrospray ionisation mass spectrometry spectral analyses as follows: nine chlorogenic acids (**1–9**), eight flavones, flavone glycosides, dihydroflavones (**10–17**), five phenylpropanoids (**18–22**), six phenolic acids (**23–28**), three caffeic acids (**29–31**), stilbene (**32**), biphenyltetrol (**33**), three phenylethanoid glycosides (**34**–**36**), hexenyl glucoside (**37**), a triterpenoid (**38**), and a steroid (**39**) (for ^1^H and ^13 ^C data of compounds **1**–**39**, see Supporting information S1). The relative and absolute configurations of compound **18** were determined by 2D-NMR and circular dichroism spectra, respectively (see Supporting information S2). Compound **18** was identified as an arylnaphthalene lignan glycoside with abnormal chirality of 8*S,*7'*S,*8'*S* absolute configurations, which was recently reported in only one case of *in vitro* bioactivity screening^26^.

### Inhibitory activity on sEH and Structure-Activity relationships (SARs)

The sEH inhibitory activities of the isolated compounds (**1**–**39**) were evaluated using a fluorescent probe based on hydrolysis of the specific substrate PHOME in the presence of sEH enzyme. 12–(3-Adamantan-1-yl-ureido) dodecanoic acid (AUDA) was used as a positive control (50% inhibitory concentration, IC_50_ = 13.3 ± 0.8 µM). Compounds **1**–**39** were tested at a concentration of 100 µM on sEH (Table 1). Sixteen compounds (**1**, **3–8**, **10**, **12**, **14–17**, **19**, **30**, and **32**) exhibited sEH inhibitory activity greater than 50% and were further examined at various concentrations. The IC_50_ value was calculated using a dose-dependent response curve, as shown in Table 1. Sixteen compounds displayed different inhibitory activities on sEH, with IC_50_ values ranging from 4.5 ± 0.2 to 60.7 ± 1.9 µM. Among them, compounds **8**, **10**, **12**, **16**, **17**, **19**, and **32** exhibited strong inhibitory activity on sEH, with IC_50_ values of 9.4 ± 0.2, 6.8 ± 2.4, 7.2 ± 0.3, 6.2 ± 0.1, 9.5 ± 0.1, 4.5 ± 0.2, and 6.8 ± 0.9 µM, respectively, relative to the positive control, AUDA (13.3 ± 0.8 µM). In addition, the lignan glycoside **18** exhibited weak inhibitory activity against sEH, although it was recently reported to induce remarkable transcriptional activation of X-box binding protein 1, which is related to ulcerative colitis^26^.

**Table 1.** Inhibition and IC_50_ values of compounds **(1–39)** on sEH^a^.

Compounds	100 µM (%)	IC_50_ (µM)	Type (Ki, µM)	Compounds	100 µM (%)	IC_50_ (µM)	Type (Ki, µM)
**1**	74.3 ± 3.4	41.9 ± 1.2	N.T	**21**	N.I	N.T	N.T
**2**	4.4 ± 3.2	N.T	N.T	**22**	31.9 ± 4.5	N.T	N.T
**3**	67.1 ± 3.4	60.7 ± 1.9	N.T	**23**	10.7 ± 3.0	N.T	N.T
**4**	68.3 ± 3.4	30.4 ± 0.8	N.T	**24**	38.6 ± 0.2	N.T	N.T
**5**	77.7 ± 2.6	33.1 ± 0.3	N.T	**25**	42.0 ± 3.9	N.T	N.T
**6**	111.1 ± 1.9	14.7 ± 0.4	N.T	**26**	21.3 ± 0.2	N.T	N.T
**7**	69.2 ± 0.9	50.8 ± 1.5	N.T	**27**	17.5 ± 0.2	N.T	N.T
**8**	165.3 ± 3.5	9.4 ± 0.2	Mixed (13.7 ± 0.2)	**28**	22.1 ± 0.9	N.T	N.T
**9**	32.3 ± 2.7	N.T	N.T	**29**	39.7 ± 4.4	N.T	N.T
**10**	58.0 ± 2.0	6.8 ± 2.4	Non-competitive (18.6 ± 0.9)	**30**	149.1 ± 4.7	21.0 ± 0.4	N.T
**11**	34.5 ± 2.5	N.T	N.T	**31**	43.0 ± 1.1	N.T	N.T
**12**	92.4 ± 2.0	7.2 ± 0.3	Non-competitive (24.4 ± 0.7)	**32**	154.9 ± 1.7	6.8 ± 0.9	N.T
**13**	22.7 ± 3.9	N.T	N.T	**33**	19.4 ± 0.8	N.T	N.T
**14**	78.0 ± 1.0	21.3 ± 0.3	N.T	**34**	13.3 ± 0.2	N.T	N.T
**15**	60.2 ± 0.5	18.3 ± 0.2	N.T	**35**	10.5 ± 3.0	N.T	N.T
**16**	95.2 ± 0.2	6.2 ± 0.1	N.T	**36**	N.I	N.T	N.T
**17**	83.8 ± 3.7	9.5 ± 0.1	N.T	**37**	24.6 ± 3.1	N.T	N.T
**18**	52.3 ± 2.6	N.T	N.T	**38**	39.5 ± 4.1	N.T	N.T
**19**	>100	4.5 ± 0.2	Mixed (10.2 ± 0.2)	**39**	N.I	N.T	N.T
**20**	N.I	N.T	N.T				
**AUDA^b^**		13.3 ± 0.8	N.T	**AUDA^b^**		13.3 ± 0.8	

N.T: Not Tested; N.I: Not Inhibition.

^a^Compounds were tested three times.

^b^AUDA was the positive control.

**Figure 3.**
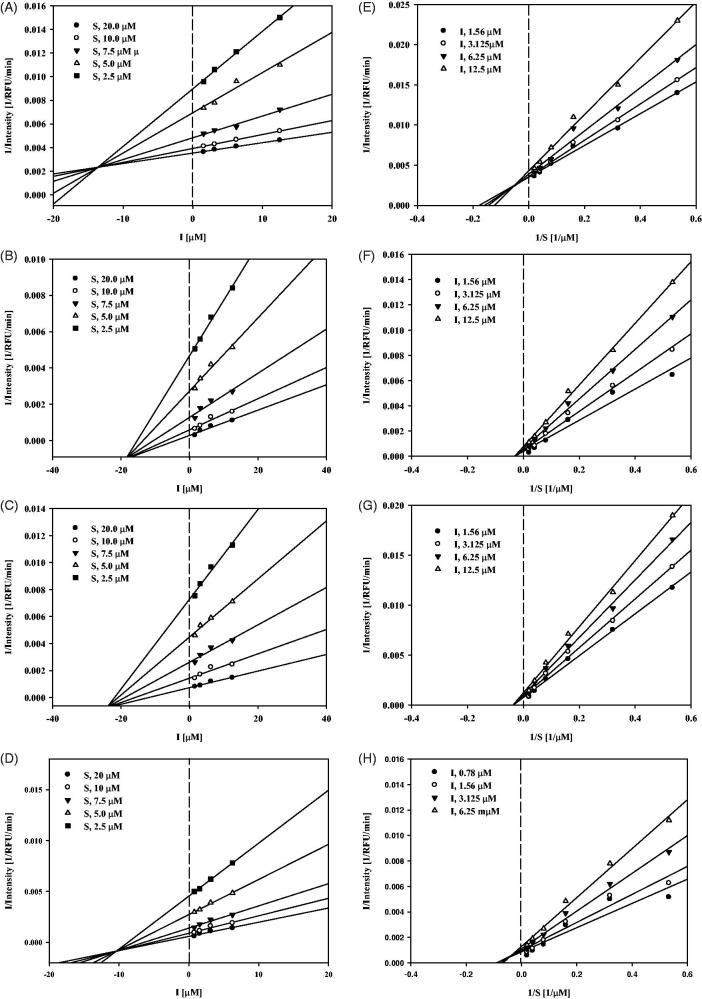
Study of binding mechanism between compounds **8**, **10**, **12**, **19** and sEH: Dixon plots for compounds **8** (A), **10** (B), **12** (C) and **19** (D), respectively. Lineweaver–Burk plots for compounds **8** (E), **10** (F), **12** (G) and **19** (H), respectively. Data are the mean of three experiments carried out in triplicate and were determined by one-way analysis of variance followed by Dunnett’s multiple comparison test, *p* < .05 versus control.

To determine the binding mechanism between seven compounds (**8**, **10**, **12**, **16**, **17**, **19**, and **32**) and sEH, their activities were measured using different substrate and inhibitor concentrations. Compounds **10** and **12** inhibited sEH activity in a non-competitive manner; compounds **8** and **19** inhibited sEH activity in a mixed manner because the different substrate concentrations resulted in a family of lines that intersected on the *x*-axis in Dixon plots (Figure 3).

Regarding the SAR analysis, the activities of the tested compounds correlated well with their ability to inhibit sEH, with activities dependent on the substituents at the R_1_ and R_2_ positions of the phenol groups or the flexible ring. The results are summarised as follows:

1. For caffeic acids and chlorogenic acids, the activities were as follows: (i) 3, 4-OH > 3-OCH_3_, 4-OH > 4-OH (of phenol groups): compounds **1** and **30**, with 3,4-dihydroxyl substitution, were more active than compounds **3** and **31**, with 3-methoxy or 4-hydroxyl substitution, while the 4-hydroxyl substituent compounds (**2** and **29**) were the worst (e.g. **1** [IC_50_ = 41.9 ± 1.2 µM] vs. **3** [IC_50_ = 60.7 ± 1.9 µM] vs. **2**; **30** [IC_50_ = 21.0 ± 0.4 µM] vs. **31** vs. **29**; (ii) –COOCH_3_ (R) > –COOH [at the flexible rings]); substitution of a methyl (hydrocarbyl) group on the carboxyl group (**8** and **4**) resulted in more activity than an unsubstituted carboxyl group (**7** and **3**) (e.g. **8** [IC_50_ = 9.4 ± 0.2 µM] vs. **7** [IC_50_ = 50.8 ± 1.5 µM]; **4** [IC_50_ = 30.4 ± 0.8 µM] vs. **3** [IC_50_ = 60.7 ± 1.9 µM]). The chirality of the six-membered ring also affected the inhibitory activities of chlorogenic acids (e.g. **4** [IC_50_ = 30.4 ± 0.8 µM] vs. **6** [IC_50_ = 14.7 ± 0.4 µM] vs. **8** [IC_50_ = 9.4 ± 0.2 µM]).
2. For flavone glycosides and their aglycones, the activities were as follows: (i) flavones > dihydroflavones: flavones (**14**) were more active than dihydroflavones (**13**) (e.g. **14** [IC_50_ = 21.3 ± 0.3 µM] vs. **13**); (ii) 3, 4-OH > 4-OH of phenyl groups: compound **16**, with 3,4-hydroxyl substitution, was more active than compound **15**, with 4-hydroxyl substitution (e.g. **16** [IC_50_ = 6.2 ± 0.1 µM] vs. **15** [IC_50_ = 18.3 ± 0.2 µM]); (iii) aglycone > glycoside: the activity of the flavonoid (**10**) was higher than that of its flavonoid glycoside (**14**) (e.g. **10** [IC_50_ = 6.8 ± 2.4 µM] vs. **14** [IC_50_ = 21.3 ± 0.3 µM]).

In summary, compounds with strong inhibitory activity against sEH tend to possess a benzene ring with dihydroxyl substituents. These results suggest that the number of OH groups in the benzene ring of the tested compounds may contribute to the increase in sEH inhibitory activity. Thus far, the main inhibitors of sEH are chlorogenic acids and flavonoids, which have been described as potent, stable, and water-soluble. In the future, good water solubility, fast gastric absorption, and long duration of action will be important in the development of new sEH inhibitors.

Flavonoids are less active than urea-based inhibitors, but their water solubility, safety, and bioavailability are highly advantageous. Their wide distribution in natural resources also provides great accessibility and promising prospects.

The results obtained in this work contribute to a better understanding of the pharmacological activities of *T*. *hemsleyanum* and its potential application values as a functional food.

### NO production in LPS-stimulated RAW 264.7 cells

The effects of all isolated compounds (**1–39**) on LPS-induced production of the inflammatory mediator NO by RAW 264.7 macrophages were evaluated (Table 2). Cell viability was assessed by determining mitochondrial reductase function using an assay based on the reduction of MTT into formazan crystals. The formation of formazan from MTT is proportional to the number of functional mitochondria in living cells. Compounds **1**–**39** did not exhibit significant cytotoxicity in RAW 264.7 cells at the evaluated concentrations (see Supporting information S3). Thus, the effects of compounds **1**–**39** on LPS-induced production of the inflammatory mediator NO in RAW 264.7 cells were evaluated at concentrations less than 50.0 µM. Bacterial LPS, either alone or in combination with interferon, is the best-characterised stimulus for the induction of inflammatory mediators, cytokines, and oxygen and nitrogen species. NO production was measured using Griess reaction assays.

**Table 2.** Inhibition of NO production in macrophage RAW264.7 cells by compounds **1**–**39** ^a^.

Compounds	50 µM(%)	IC_50_ (µM)	Compounds	50 µM(%)	IC_50_ (µM)
**1**	5.7	N.T	**21**	16.2	N.T
**2**	19.7	N.T	**22**	18.3	N.T
**3**	26.1	N.T	**23**	14.1	N.T
**4**	N.I	N.T	**24**	22.2	N.T
**5**	53.0	45.3 ± 1.4	**25**	17.0	N.T
**6**	1.8	N.T	**26**	11.4	N.T
**7**	10.7	N.T	**27**	24.0	N.T
**8**	25.2	N.T	**28**	6.2	N.T
**9**	N.I	N.T	**29**	13.4	N.T
**10**	93.0	15.6 ± 1.6	**30**	21.5	N.T
**11**	10.8	N.T	**31**	10.6	N.T
**12**	98.9	12.2 ± 0.1	**32**	77.7	30.6 ± 1.8
**13**	N.I	N.T	**33**	3.0	N.T
**14**	9.0	N.T	**34**	12.8	N.T
**15**	3.0	N.T	**35**	15.9	N.T
**16**	26.2	N.T	**36**	20.9	N.T
**17**	29.9	N.T	**37**	14.5	N.T
**18**	32.1	N.T	**38**	8.9	N.T
**19**	50.1	47.3 ± 6.9	**39**	20.3	N.T
**20**	13.9	N.T			
**Dexamethasone^b^**		10.5 ± 0.1	**Dexamethasone^b^**		10.5 ± 0.1

N.T: Not Tested; N.I: Not Inhibition.

^a^Data were shown as IC_50_ values (means ± SE) of experiments performed in triplicate (*n* = 3).

TH extracts were exhibited IC_50_ concentration at 22.69 ± 0.75 µM.

^b^Dexametasone was used as the positive control.

Each compound was screened at concentrations of 25.0 and 50.0 µM. At 50.0 µM, compounds **5**, **10**, **12**, and **32** exhibited potent inhibitory activities, with inhibition values of 53.0%, 93.0%, 98.9%, and 77.7%, respectively, and IC_50_ values of 45.3 ± 1.38, 15.6 ± 1.57, 12.2 ± 0.09, 47.3 ± 6.88, 47.2, and 30.6 ± 1.80 µM, respectively, relative to the positive control, dexamethasone (10.53 ± 0.13 µM) (Table 2).

SAR assays of compounds **1**–**39** revealed flavone (**10**) exerted stronger inhibitory activity against NO production than its flavone glycoside (**14**) and dihydroflavonoid aglycone (**13**). These results indicate that the sugar unit in the flavone glycoside is unfavourable for efficient inhibition of NO production.

### Molecular modeling

**Figure 4.**
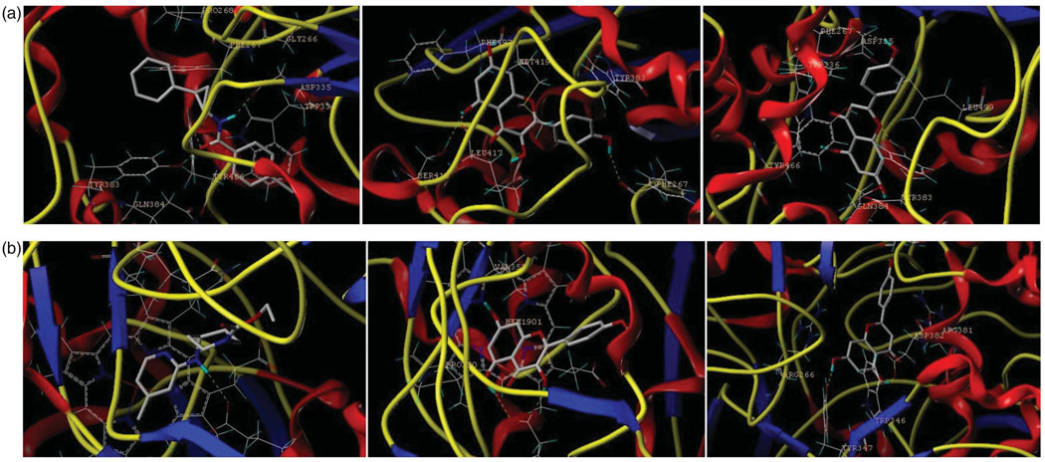
The binding model of compounds **10** and **12** in complex with sEH and iNOS. (a) The proposed binding mode and interaction of molecular modelling between sEH and A/S38_601, compounds **10** and **12**. (b) The proposed binding mode and interaction of molecular modelling between iNOS and AT2_1906, compounds **10** and **12**. The compounds and important amino acids in the binding pockets are shown in stick model, whereas sEH is depicted in the ribbon model.

All docking studies were carried out using Sybyl-X 2.0 on a Windows workstation (Figure 4). The crystal structures of sEH (PDB: 3ans.pdb)^25^ and iNOS (PDB: 3e7g.pdb)^27^ were retrieved from the RCSB Protein Data Bank. The isolated compounds were selected for docking studies. The three-dimensional structures of the selected compounds were first built using Sybyl-X 2.0 sketch followed by energy minimisation using the MMFF94 force field and Gasteiger-Marsili charges. The geometry was optimised with a distance-dependent dielectric constant and a termination energy gradient of 0.001 kcal/mol employed by Powell’s method. All of the selected compounds were automatically docked into the ligand-binding pocket of tubulin by an empirical scoring function and a patented search engine in the Surflex docking programme. Before the docking process, the natural ligand (sEH: A/S38_601; iNOS: AT2_1906) was extracted and the water molecules were removed from the crystal structure. Subsequently, the protein was prepared using the biopolymer module implemented in Sybyl. Polar hydrogen atoms were added. The automated docking manner was applied. The optimal binding pose of the docked compounds was selected based on the consensus scoring schemes and visual inspection of the docked complexes (see Supporting information S4–S8).

## Conclusion

In conclusion, 35 compounds were isolated from *T*. *hemsleyanum* for the first time. Among them, two flavonoids, kaempferol (**10**) and apigenin (**12**), exhibited remarkable inhibitory activities against sEH and iNOS. These results indicate that **10** and **12** may act as dual inhibitors of sEH and iNOS. This study provides evidence for the contribution of constituents to the special pharmacological activities of *T*. *hemsleyanum* as a functional food.

## Supplementary Material

Supplemental Material
