# Supplementary material for: Inhibition potential of phenolic constituents from the aerial parts of Tetrastigma hemsleyanum against soluble epoxide hydrolase and nitric oxide synthase
Source: J Enzyme Inhib Med Chem. 2019 Mar 4;34(1):753–60. doi: 10.1080/14756366.2019.1584621 (PMC6407587; doi:10.1080/14756366.2019.1584621)
Supplement: Supplemental Material [file IENZ_A_1584621_SM1530.pdf]

Inhibition potential of phenolic constituents from the aerial parts of *Tetrastigma hemsleyanum* against soluble epoxide hydrolase and nitric oxide synthase

Cai Yi Wang [a, 1], Sunggun Lee [a, 1], Hyun-Jae Jang [b], Xiang Dong Su [a], Heng-Shan Wang [c], Young Ho Kim [a, *] and Seo Young Yang [a, *]

[a] *College of Pharmacy, Chungnam National University, Daejeon 34134, Republic of Korea;*

[b] *Immunoregulatory Material Research Center, Korea Research Institute of Bioscience and Biotechnology, 181 Ipsin-gil, Jeongeup-si, Jeonbuk 56212, Korea*

[c] *State Key Laboratory for Chemistry and Molecular Engineering of Medicinal Resources, School of Chemistry and Pharmaceutical Sciences, Guangxi Normal University, Guilin 541004, People's Republic of China*

*Correspondence:

Dr. Young Ho Kim, College of Pharmacy, Chungnam National University, Daejeon 34134, Republic of Korea.

Tel: 82-42-821-5933 Fax: 82-42-823-6566 E-mail: yhk@cnu.ac.kr

Dr. Seo Young Yang, College of Pharmacy, Chungnam National University, Daejeon 34134, Republic of Korea.

Tel: 82-42-821-7321 Fax: 82-42-823-6566 E-mail: syyang@cnu.ac.kr

Contents

S1. ^{1}H NMR and ^{13}C NMR data of compounds 1-39 --3

S2. Structure elucidation of compound 18 --16

Figure S1. HMBC, COSY correlations of compound 18 --17

Table S1. Comparing the data ^{1}HNMR and ^{13}CNMR data of compound 18 with reference ----------------------------------18

Figure S2. CD spectrum of compound 18 --19

Figure S3. ^{1}H NMR spectrum of compound 18 in CD_3OD (400 MHz) --20

Figure S4. ^{13}C NMR spectrum of compound 18 in CD_3OD (100 MHz) --21

Figure S5. HMQC spectrum of compound 18 in CD_3OD (400 MHz) -- 22

Figure S6. HMBC spectrum of compound 18 in CD_3OD (400 MHz) -- 23

Figure S7. COSY spectrum of compound 18 in CD_3OD (400 MHz) -- 24

Figure S8. NOESY spectrum of compound 18 in CD_3OD (400 MHz) -- 25

S3. MTT assay data of compounds-- 26

S4. Interaction of molecular modeling between sEH and A/S38_601, compound 10, 12 -- 27

S5. Interaction of molecular modeling between iNOS and AT2_1906, compound 10, 12-- 28

References-- 29

S1. ^{1}H NMR and ^{13}C NMR data of compound 1–39

*(1R,3R,4S,5R)-3-O-(E)-Caffeoylquinic acid (**1**)* (dos Santos et al., 2004)

^{1}H NMR (400 MHz, CD_3OD) δ 7.59 (d, *J* = 15.9 Hz, 1H), 7.05 (d, *J* = 1.8 Hz, 1H), 6.94 (dd, *J* = 8.2, 1.8 Hz, 1H), 6.80 – 6.73 (m, 1H), 6.30 (t, *J* = 13.2 Hz, 1H), 5.36 (d, *J* = 3.6 Hz, 1H), 4.24 – 4.06 (m, 1H), 3.65 (dd, *J* = 8.4, 3.2 Hz, 1H), 2.28 – 2.03 (m, 3H), 1.96 (dd, *J* = 13.4, 9.7 Hz, 1H).^{13}C NMR (100 MHz, CD_3OD) δ178.7, 169.2, 149.6, 146.9, 128.0, 123.0, 116.52, 115.9,115.1 75.4, 74.7, 73.0, 68.4, 41.4, 36.7.

*(1R,3R,4S,5R)-3-O-(E)-Coumaroylquinic acid (**2**)* (Kuczkowiak et al., 2014)

^{1}H NMR (400 MHz, CD_3OD) δ 7.65 (d, *J* = 15.9 Hz, 1H), 7.46 (d, *J* = 8.6 Hz, 2H), 6.80 (d, *J* = 8.6 Hz, 2H), 6.37 (d, *J* = 15.9 Hz, 1H), 5.36 (dd, *J* = 7.3, 3.6 Hz, 1H), 4.16 (td, *J* = 9.4, 4.1 Hz, 1H), 3.64 (dd, *J* = 8.5, 3.3 Hz, 1H), 2.18 (dd, *J* = 12.6, 3.6 Hz, 3H), 1.96 (dd, *J* = 13.3, 10.0 Hz, 1H). ^{13}C NMR (100 MHz, CD_3OD) δ 178.3, 169.0, 161.1, 146.4, 131.1, 127.4, 116.8, 115.9, 75.4, 74.8, 73.0, 68.3, 41.5, 36.7

*(1R,3R,4S,5R)-3-O-(E)-Feruloylquinic acid (**3**)* (Iwai et al., 2004)

^{1}H NMR (400 MHz, CD_3OD) δ 7.65 (d, *J* = 15.9 Hz, 1H), 7.20 (d, *J* = 1.8 Hz, 1H), 7.06 (dt, *J* = 20.2, 10.1 Hz, 1H), 6.82 (t, *J* = 9.8 Hz, 1H), 6.39 (dd, *J* = 18.8, 9.9 Hz, 1H), 5.36 (d, *J* = 3.7 Hz, 1H), 4.20 – 4.10 (m, 1H), 3.88 (d, *J* = 11.9 Hz, 3H), 3.70 – 3.60 (m, 1H), 2.26 – 2.09 (m, 3H), 2.03 – 1.91 (m, 1H). ^{13}C NMR (100 MHz, CD_3OD) δ 178.18, 167.31, 150.15, 148.74, 145.88, 127.60, 124.09, 116.14, 116.14, 111.40, 75.36, 74.54, 72.80, 68.41, 56.47, 40.95, 36.24

*(1R,3R,4S,5R)-5-O-(E)-Caffeoylquinic acid methyl ester (**4**)* (Zhu et al., 2005)

^{1}H NMR (400 MHz, CD_3OD) δ 7.59 (d, *J* = 15.9 Hz, 1H), 7.05 (d, *J* = 1.8 Hz, 1H), 6.95 (dd, *J* = 8.2, 1.6 Hz, 1H), 6.78 (d, *J* = 8.2 Hz, 1H), 6.31 (d, *J* = 15.9 Hz, 1H), 5.36 (dd, *J* = 9.0, 3.6 Hz, 1H), 4.13 (td, *J* = 8.3, 3.9 Hz, 1H), 3.73 (s, 3H), 3.69 (dd, *J* = 7.8, 3.3 Hz, 1H), 3.31 (s, 1H), 2.21 (dd, *J* = 14.3, 3.8 Hz, 1H), 2.16 – 2.12 (m, 1H), 2.10 (d, *J* = 3.8 Hz, 1H), 2.00 (dd, *J* = 13.4, 8.8 Hz, 1H). ^{13}C NMR (100 MHz, CD_3OD) δ 176.5, 169.0, 149.4, 146.8, 146.7, 127.9, 122.9, 116.5, 115.7, 115.1, 75.3, 73.9, 72.6, 68.5, 52.9, 40.8, 36.3.

*(1R,3R,4S,5R)-5-O-(E)- Caffeoylquinic acid butyl ester (**5**)* (Zhu et al., 2010)

^{1}H NMR (400 MHz, CD_3OD) δ 7.59 (d, *J* = 15.9 Hz, 1H), 7.04 (t, *J* = 7.9 Hz, 1H), 6.95 (dd, *J* = 8.2, 2.0 Hz, 1H), 6.78 (d, *J* = 8.2 Hz, 1H), 6.31 (d, *J* = 15.9 Hz, 1H), 5.37 (dd, *J* = 9.0, 3.6 Hz, 1H), 4.24 – 4.05 (m, 3H), 3.64 (ddd, *J* = 21.1, 11.0, 5.2 Hz, 2H), 2.27 – 1.91 (m, 4H), 1.65 (tt, *J* = 20.9, 10.5 Hz, 2H), 1.42 (dt, *J* = 14.6, 7.4 Hz, 2H), 1.16 (dd, *J* = 17.3, 10.2 Hz, 1H), 0.95 (t, *J* = 7.4 Hz, 3H). ^{13}C NMR (100 MHz, CD_3OD) δ 176.1, 169.0, 149.4, 146.8, 146.8, 127.9, 122.9, 116.4, 115.7, 115.1, 75.4, 74.0, 72.7, 68.5, 66.3, 40.9,

36.4, 31.7, 20.1, 14.0.

*(1S,3R,4R,5R)-3-O-(E)-Caffeoylquinic acid (**6**)* (Zhu et al., 2005)

^{1}H NMR (400 MHz, CD_3OD) δ 7.51 (d, *J* = 15.9 Hz, 1H), 7.03 (d, *J* = 2.1 Hz, 1H), 6.93 (dd, *J* = 8.2, 2.1 Hz, 1H), 6.77 (dd, *J* = 7.7, 4.1 Hz, 1H), 6.20 (d, *J* = 15.9 Hz, 1H), 5.27 (td, *J* = 7.8, 4.4 Hz, 1H), 4.13 (dd, *J* = 6.6, 3.4 Hz, 1H), 3.73 (dd, *J* = 7.6, 3.2 Hz, 1H), 3.68 (s, 3H), 2.19 (dd, *J* = 13.7, 3.7 Hz, 2H), 2.11 (dd, *J* = 13.4, 8.2 Hz, 1H), 2.00 (dd, *J* = 13.4, 6.0 Hz, 1H). ^{13}C NMR (100 MHz, CD_3OD) δ 175.4, 168.3, 149.6, 147.2, 146.7, 127.6, 123.0, 116.5, 115.1, 115.0, 75.8, 72.5, 72.0, 70.3, 53.0, 40.2, 37.9.

*(1α,3R,4α,5R)-4-O-(E)-Caffeoylquinic acid (**7**)* (Iwai et al., 2004)

^{1}H NMR (400 MHz, CD_3OD) δ 7.64 (d, *J* = 15.9 Hz, 1H), 7.09 (t, *J* = 12.2 Hz, 1H), 6.97 (dd, *J* = 8.2, 1.8 Hz, 1H), 6.77 (t, *J* = 11.0 Hz, 1H), 6.37 (d, *J* = 15.9 Hz, 1H), 4.80 (d, *J* = 6.9 Hz, 1H), 4.30 (s, 1H), 4.26 (m, 1H), 2.31 – 2.14 (m, 2H), 2.13 – 1.92 (m, 2H). ^{13}C NMR (100 MHz, CD_3OD) δ 169.1, 149.6, 147.2, 146.8, 127.8, 123.0, 116.5, 115.3, 115.2, 79.3, 69.6, 65.6, 42.7, 38.4.

*(1α,3R,4α,5R)-4-O-(E)-Caffeoylquinic acid methyl ester (**8**)* (Zhu et al., 2005)

^{1}H NMR (400 MHz, CD_3OD) δ 7.64 (dd, *J* = 15.9, 3.1 Hz, 1H), 7.07 (d, *J* = 2.0 Hz, 1H), 6.97 (dd, *J* = 8.2, 2.0 Hz, 1H), 6.79 (d, *J* = 8.2 Hz, 1H), 6.37 (dd, *J* = 15.9, 3.9 Hz, 1H), 4.79 – 4.71 (m, 1H), 4.29 (dt, *J* = 9.4, 3.1 Hz, 1H), 4.25 (m, 1H), 3.75 (s, 3H), 2.23 – 2.14 (m, 2H), 2.09 – 1.98 (m, 2H). ^{13}C NMR (100 MHz, CD_3OD) δ 175.7, 169.0, 149.5, 147.1, 146.8, 127.8, 123.0, 116.5, 115.3, 115.2,

78.6, 76.4, 69.0, 65.7, 53.0, 42.1, 38.4.

*(1R,3R,4S,5R)-3-O-(Z)-Coumaroylquinic acid (**9**)* (Tanaka et al., 2014)

^{1}H NMR (400 MHz, CD_3OD) δ 7.78 – 7.62 (m, 2H), 6.83 (dd, *J* = 11.9, 4.7 Hz, 1H), 6.79 – 6.69 (m, 2H), 5.84 (t, *J* = 10.5 Hz, 1H), 5.32 (dd, *J* = 8.2, 3.7 Hz, 1H), 4.08 (td, *J* = 8.7, 4.2 Hz, 1H), 3.71 – 3.60 (m, 1H), 2.25 – 2.05 (m, 3H), 2.03 – 1.89 (m, 1H). ^{13}C NMR (100 MHz, CD_3OD) δ 178.7, 167.8, 160.0, 144.9, 133.8, 127.7, 117.2, 115.7, 75.6, 74.7, 72.7, 68.5, 36.6, 30.2.

*Kaempferol (**10**)* (Itoh et al., 2009)

^{1}H NMR (400 MHz, CD_3OD) δ 8.09 (d, *J* = 8.9 Hz, 2H), 6.90 (d, *J* = 8.9 Hz, 2H), 6.39 (d, *J* = 2.1 Hz, 1H), 6.18 (d, *J* = 2.0 Hz, 1H). ^{13}C NMR (100 MHz, CD_3OD) δ 177.5, 165.7, 162.6, 160.7, 158.4, 148.2, 130.8, 123.8, 116.4, 104.6, 99.3, 94.5.

*(2R-trans)-3,4',7-Trihydroxyflavanone (**11**)* (Blanco et al., 2007)

ESI-MS m/z: 206.05 $[M-H]^-$; ^{1}H NMR (400 MHz, CD_3OD) δ 7.74 (d, *J* = 8.7 Hz, 1H), 7.33 (d, *J* = 8.5 Hz, 2H), 6.82 (d, *J* = 8.6 Hz, 2H), 6.50 (dd, *J* = 8.7, 2.3 Hz, 1H), 6.36 (d, *J* = 2.3 Hz, 1H), 5.39 (dd, *J* = 13.1, 2.9 Hz, 1H), 2.70 (dd, *J* = 16.9, 2.9 Hz, 1H).

*Apigenine (**12**)* [12] (Nagao et al., 2002)

ESI-MS m/z: 271.03 $[M+H]^+$; ^{1}H NMR (400 MHz, CD_3OD) δ 7.85 (d, *J* = 8.8 Hz, 2H), 6.93 (d, *J* = 8.8 Hz, 2H), 6.59 (s, 1H), 6.45 (d,

J = 2.1 Hz, 1H), 6.21 (d, *J* = 2.1 Hz, 1H), 4.61 (s, 2H), 1.38 – 1.18 (m, 6H).

*(2R,3R)-Dihydrokaempferol 3-O-β-D-glucoside (**13**)* (Godecke et al., 2005)

^{1}H NMR (400 MHz, CD_3OD) δ 7.36 (d, *J* = 8.3 Hz, 1H), 6.82 (d, *J* = 8.3 Hz, 1H), 5.91 (d, *J* = 7.4 Hz, 1H), 5.28 (d, *J* = 10.1 Hz, 1H), 4.98 (d, *J* = 10.1 Hz, 1H). ^{13}C NMR (101 MHz, CD_3OD) δ 195.1, 168.0, 164.5, 163.2, 158.3, 129.5, 127.5, 115.2, 101.6, 96.3, 95.3, 82.5, 77.2, 76.5, 76.2, 73.5, 70.2, 61.5.

*Kaempferol 3-O-β-D-glucopyranoside (**14**)* (Chae et al., 2002)

^{1}H NMR (400 MHz, DMSO-*d6*) δ 12.63 (s, 1H), 10.23 (s, 1H), 8.03 (d, 2H, *J*=8.7 Hz), 6.89 – 6.80 (d, 2H, *J*=8.7 Hz), 6.40 (d, *J* = 2.0 Hz, 1H), 6.20 (d, *J* = 2.0 Hz, 1H), 5.46 (d, *J* = 7.5 Hz, 1H), 3.58 (d, *J* = 11.4 Hz, 2H), 3.28 – 3.19 (m, 4H), 3.09 (d, *J* = 3.6 Hz, 3H), 1.22 (s, 1H). ^{13}C NMR (100 MHz, DMSO-*d6*) δ 177.5, 164.2, 161.3,160.4 156.4, 156.2, 133.3, 131.3, 121.2, 115.2, 104.0, 100.9, 98.7, 93.5, 77.6, 76.5, 74.1, 70.0, 61.0.

*3-[[6-O-(6-Deoxy-α-L-mannopyranosyl)-β-D-galactopyranosyl]oxy]-5,7-dihydroxy-2-(4-hydroxyphenyl)-4H-1-benzopyran-4-one (**15**)* (Markham et al., 1978)

^{1}H NMR (400 MHz, DMSO-*d6*) δ 12.57 (d, *J* = 1.4 Hz, 1H), 10.86 (s, 1H), 10.14 (s, 1H), 7.98 (dd, *J* = 8.7, 1.6 Hz, 2H), 6.88 (dd, *J* = 8.7, 1.7 Hz, 2H), 6.45 – 6.38 (m, 1H), 6.20 (t, *J* = 2.0 Hz, 1H), 5.36 (d, *J* = 4.0 Hz, 1H), 5.31 (d, *J* = 7.3 Hz, 1H), 5.10 (dd, *J* = 10.6,

5.0 Hz, 2H), 4.56 (d, *J* = 4.4 Hz, 1H), 4.43 (dd, *J* = 8.8, 5.0 Hz, 2H), 4.37 (s, 1H), 3.68 (d, *J* = 10.1 Hz, 1H), 3.41 (s, 2H), 3.32 – 3.20 (m, 5H), 3.15 (dd, *J* = 11.7, 5.6 Hz, 2H), 3.12 – 2.99 (m, 2H), 0.98 (d, *J* = 5.9 Hz, 3H). ^{13}C NMR (100 MHz, DMSO-*d*6) δ 177.5, 164.3, 161.3, 160.0, 157.0, 156.6, 133.3, 131.0, 121.0, 115.2, 104.1, 101.4, 100.8, 98.8, 93.8, 76.4, 75.8, 74.2, 71.8, 70.6, 70.4, 70.0, 68.3, 66.9, 48.6, 17.7.

*3-[[6-O-(6-Deoxy-α-L-mannopyranosyl)-β-D-glucopyranosyl]oxy]-2-(3,4-dihydroxyphenyl)-5,7-dihydroxy-4H-1-benzopyran-4-one (**16**)* (Xiao et al., 2005)

^{1}H NMR (400 MHz, DMSO-*d*6) δ 7.55 (t, *J* = 2.8 Hz, 1H), 7.53 (s, 1H), 6.89 – 6.79 (m, 1H), 6.37 (t, *J* = 6.9 Hz, 1H), 6.19 (d, *J* = 1.7 Hz, 1H), 5.33 (dd, *J* = 10.1, 6.7 Hz, 2H), 5.12 (d, *J* = 9.4 Hz, 2H), 4.39 (d, *J* = 14.5 Hz, 2H), 3.79 – 3.62 (m, 2H), 3.24 (ddd, *J* = 21.1, 16.7, 8.2 Hz, 8H), 3.16 (s, 1H), 3.07 (t, *J* = 9.2 Hz, 2H), 2.50 (s, 4H), 0.99 (d, *J* = 6.1 Hz, 3H). ^{13}C NMR (100 MHz, DMSO-*d*6) δ 177.5, 164.3, 161.4, 156.8, 156.6, 148.6, 144.9, 133.4, 121.7, 121.3, 116.4, 115.3, 104.0, 101.3, 100.8, 98.8, 93.7, 76.5, 76.0, 74.1, 71.9, 70.6, 70.4, 70.0, 68.3, 67.0, 48.6, 17.7.

*3-[[6-O-(2-O-acetyl-6-deoxy-α-L-mannopyranosyl)-β-D-glucopyranosyl]oxy]-2-(3,4-dihydroxyphenyl)-5,7-dihydroxy-4H-1-Benzopyran-4-one (**17**)* (Kasper et al., 2010)

^{1}H NMR (400 MHz, DMSO-*d*6) δ 7.65 – 7.61 (m, 2H), 6.86 – 6.81 (m, 1H), 6.37 (d, *J* = 1.6 Hz, 1H), 6.18 (d, *J* = 1.6 Hz, 1H), 5.16 (d, *J* = 7.5 Hz, 1H), 4.54 (dd, *J* = 9.7, 3.1 Hz, 1H), 4.45 (s, 1H), 4.40 (s, 1H), 3.85 – 3.69 (m, 4H), 3.51 (m, 2H), 3.32 – 3.25 (m, 1H), 3.25

– 3.18 (m, 1H), 1.97 (s, 3H), 1.02 (d, *J* = 6.0 Hz, 3H). ^{13}C NMR (100 MHz, DMSO-d6) δ 177.5, 170.0, 164.2, 161.4, 156.7, 156.5, 148.5, 144.8, 133.4, 121.6, 121.3, 116.3, 115.3, 104.0, 101.3, 100.8, 79.1, 76.5, 75.9, 74.2, 74.1, 70.2, 68.8, 68.4, 67.7, 21.0, 17.6.

*(-)-(8S,7'R,8'S)-Burselignan-9'-O-a-L-rhamnoside (**18**)* (Zhou et al., 2016)

$C_{26}H_{34}O_{10}$, amorphous solid; ESI-MS m/z: 507.22 $[M+H]^+$; UV (MeOH) λ_{max} 203, 284 nm; ^1H NMR (400 MHz, CD_3OD) δ 6.76 (d, *J* = 8.0 Hz, 1H, H-5'), 6.67 (s, 1H, H-6), 6.64 (d, *J* = 1.8 Hz, 1H, H-2'), 6.59 (dd, *J* = 8.0, 1.9 Hz, 1H, H-6'), 6.17 (s, 1H, H-3), 4.52 (d, *J* = 1.4 Hz, 1H, H-1''), 3.87 (d, J = 10.4 Hz, 1H, H-7'), 3.83 (m, 1H, H-9'a), 3.81 (s, 3H, 5-OCH_3), 3.77 (s, 3H, 3'-OCH_3), 3.74 (m, 2H, H-9a, 2''), 3.68 – 3.60 (m, 1H, H-9b), 3.52 (m, 1H, H-3'', H-5''), 3.36 (m, 1H, H-4''), 3.12 (dd, *J* = 9.8, 3.6 Hz, 1H, H-9'b), 2.83 (d, *J* = 7.4 Hz, 1H, H-7), 2.03 (m, 1H, H-8'), 1.86 (t, *J* = 10.1 Hz, 1H, H-8), 1.19 (d, *J* = 6.2 Hz, 3H, H-6''). ^{13}C NMR (100 MHz, CD_3OD) δ 147.3 (C-3'), 146.1 (C-5), 145.2 (C-4'), 141.1 (C-4), 134.0 (C-1'), 130.0 (C-2), 128.9 (C-1), 123.1 (C-6'), 117.1 (C-3), 116.1 (C-5’), 113.5 (C-2'), 112.5 (C-6), 102.3 (C-1''), 72.5 (C-4''), 70.3 (C-3''), 70.1 (C-2''), 68.0 (C-5''), 65.4 (C-9'), 63.4 (C-9), 56.4 (C-OCH_3), 49.6 (C-7'), 45.5 (C-8), 40.1 (C-8'), 33.6 (C-7), 17.9 (C-6'').

*6,7-dihydroxycoumarin (**19**)* (Chang et al., 1977)

^1H NMR (400 MHz, CD_3OD) δ 7.78 (d, *J* = 9.4 Hz, 1H), 6.94 (s, 1H), 6.75 (s, 1H), 6.18 (d, *J* = 9.4 Hz, 1H). ^{13}C NMR (100 MHz, CD_3OD) δ 164.3, 151.9, 150.4, 146.1, 144.5, 113.0, 112.8, 112.5, 103.6.

*(7R,8S)-dihydrodehydrodiconiferyl alcohol 9-O-β-D-glucopyranoside (**20**)* (Su et al., 2008)

$C_{26}H_{34}O_{11}$, amorphous solid; ESI-MS m/z: 523.22 $[M+H]^+$; UV (MeOH) λ_{max} 209, 231, 285 nm; ^1H NMR (400 MHz, CD_3OD) δ 7.00 (d, *J* = 1.9 Hz, 1H, H-2), 6.85 (dd, *J* = 8.1, 1.9 Hz, 1H, H-6), 6.80 (s, 1H, , H-2'), 6.75 (d, *J* = 8.1 Hz, 1H, H-5), 6.72 (s, 1H, H-6'), 5.58 (d, *J* = 6.4 Hz, 1H, H-7), 4.35 (d, *J* = 7.7 Hz, 1H, H-1''), 4.11 (dd, *J* = 9.6, 8.0 Hz, 1H , H-9a), 3.89 – 3.86 (m, 1H, H-9b), 3.85 (s, 3H, OCH_3), 3.82 (s, 3H, OCH_3), 3.74 (m, 1H, H-6''a), 3.70 – 3.62 (m, 2H, H-8, H-6''b), 3.56 (t, *J* = 6.5 Hz, 1H, H-9'a), 3.29 – 3.19 (m, 4H, H-2''- H-5''), 2.54 (m, 1H, H-7'), 1.81 (m, 1H, H-8').^{13}C NMR (100 MHz, CD_3OD) δ 149.0 (C-3), 147.7 (C-4'), 147.5 (C-4), 145.2 (C-3'), 137.0 (C-1'), 134.7 (C-1), 129.7 (C-5'), 119.8 (C-6), 118.2 (C-2'), 116.1(C-5), 114.2 (C-6'), 110.8 (C-2), 104.3 (C-1''), 89.2 (C-7), 78.2 (C-3''), 78.1 (C-5''), 75.2 (C-2''), 72.3 (C-9), 71.6 (C-4''), 62.7 (C-6''), 62.2 (C-9'), 56.8 (OCH_3), 56.5 (OCH_3), 52.9 (C-8), 35.8 (C-8'), 32.9(C-7').

*(+)-7R,8S-5-Methoxydihydrodehydroconiferyl alcohol (**21**)* (Chin et al., 2008)

$C_{21}H_{26}O_7$, amorphous solid; ESI-MS m/z: 391.13 $[M+H]^+$; UV (MeOH) λmax 209, 291 nm; ^1H NMR (400 MHz, DMSO) δ 6.71 (s, 2H, H-2, 6), 6.69 (s, 2H, H-2', 6'), 5.46 (d, *J* = 6.9 Hz, 1H, H-7), 3.83 (m, 1H, H-9a), 3.79 (s, 3H, 3'-OCH_3), 3.76 (m, 1H, H-9b), 3.75 (s, 6H, 3-OCH_3, 5-OCH_3), 3.56 (m, 2H, H-9'), 2.58 – 2.52 (m, 2H, H-7'), 1.77 – 1.63 (m, 2H, H-8').^{13}C NMR (100 MHz, DMSO) δ 155.0 (C-3, 5), 147.5 (C-3'), 145.5 (C-4'), 137.4 (C-4), 137.2 (C-1, 1'), 130.9 (C-5'), 118.5 (C-6'), 114.7 (C-2'), 105.3 (C-2, 6), 88.8 (C-7), 65.0 (C-9), 62.3 (C-9'), 58.1 (C-8), 57.8 (3-OCH_3, 5-OCH_3), 55.4 (3' -OCH_3), 36.8 (C-8'), 33.6 (C-7').

*Catechin-(5, 6-bc)-4β-(3,4-dihydroxyphenyl)-dihydro-2(3H)-pyranone (**22**)* (Bai et al., 2015)

$C_{24}H_{20}O_9$, amorphous solid; ESI-MS m/z: 453.26 $[M+H]^+$; UV (MeOH) λ_{max} 230, 284 nm; ^{1}H NMR (400 MHz, CD_3OD) δ 6.83 (s, 2H), 6.77 (d, *J* = 1.9 Hz, 1H, H-2'), 6.72 (d, *J* = 8.1 Hz, 1H, , H-5'), 6.67 (dd, *J* = 8.1, 2.0 Hz, 1H, H-6'), 6.56 (d, *J* = 8.1 Hz, 1H, H-5"), 6.50 (d, *J* = 1.6 Hz, 1H, H-2'),6.47 (dd, *J* = 8.2, 2.1 Hz, 1H, H-6"), 6.23 (s, 1H, H-8), 4.71 (d, *J* = 6.9 Hz, 1H, H-2), 4.45 (d, *J* = 6.3 Hz, 1H, H-*β*), 3.08 – 2.96 (m, 1H, H-*a*), 2.96 – 2.83 (m, 2H, H-a, 4*β*), 2.75 – 2.59 (m, 1H, H-4*a*). δ 170.3 (COO-), 155.8 (C-8a), 154.8 (C-7), 151.9 (C-5), 146.3 (C-4', 3", 4"), 145.2 (C-3'), 134.9 (C-1"), 131.8 (C-1'), 119.7 (C-6'), 119.2 (C-6"), 116.5 (C-5"), 116.2 (C-5'), 115.0 (C-2'), 115.0 (C-2"), 107.2 (C-6), 101.5 (C-4*a*), 99.8 (C-8), 82.8 (C-2), 68.1 (C-3), 38.3 (C-*a*), 35.2 (C-*β*), 27.8 (C-4).

*Syringic acid (**23**)* (Chang et al., 2000)

^{1}H NMR (400 MHz, DMSO-*d*6) δ 7.31 (s, 2H), 3.89 (s, 6H). ^{13}C NMR (100 MHz, DMSO-*d*6) δ 168.7, 148.2, 140.7, 121.6, 107.5, 56.6, 49.3.

*3,4-Dihydroxybenzaldehyde (**24**)* (Pouységu et al., 2010)

^{1}H NMR (400 MHz, DMSO-*d*6) δ 9.55 (d, *J* = 7.7 Hz, 1H), 9.51 (s, 1H), 9.19 (d, *J* = 7.8 Hz, 1H). ^{13}C NMR (100 MHz, DMSO-*d*6) δ 191.4, 152.6, 146.1, 128.8, 124.8, 115.7, 114.4.

*p-Salicylic acid (**25**)* (Sarika et al., 2010)

^{1}H NMR (400 MHz, CD_3OD) δ 7.78 (d, *J* = 8.7 Hz, 1H), 6.73 (t, *J* = 8.6 Hz, 1H). ^{13}C NMR (100 MHz, CD_3OD) δ 168.7, 161.9, 131.6, 121.3, 114.6.

*Vanillic acid (**26**)* (Miyazawa et al., 2003)

^{1}H NMR (400 MHz, DMSO-*d6*) δ 7.44 (s, 1H), 7.42 (s, 1H), 6.84 (d, *J* = 8.6 Hz, 1H). ^{13}C NMR (100 MHz, DMSO-*d6*) δ 167.7, 151.1, 147.4, 123.6, 122.20, 115.1, 112.8, 55.6.

*Isovanillic acid (**27**)* (Lai et al., 1985)

^{1}H NMR (400 MHz, CD_3OD) δ 7.55 (d, *J* = 6.7 Hz, 2H), 7.56 – 7.55 (m, 1H), 6.84 (d, *J* = 8.7 Hz, 1H), 3.89 (s, 3H), 3.88 (s, 1H). ^{13}C NMR (100 MHz, CD_3OD) δ 170.0, 152.6, 148.6, 125.2, 123.0, 115.8, 113.8, 108.3, 56.8, 56.4.

*p-Hydroxyphenacyl alcohol (**28**)* (Kelvin and Youla, 1991)

^{1}H NMR (400 MHz, DMSO-*d6*) δ 7.84 – 7.76 (m, 2H), 6.85 (t, *J* = 5.7 Hz, 2H), 4.69 (s, 2H). ^{13}C NMR (150 MHz, DMSO-*d6*) δ 197.1, 162.4, 130.1, 125.6, 115.3, 99.5, 64.8, 48.6.

*4-Coumaric acid (**29**)* (Nilsson et al., 2004)

^{1}H NMR (400 MHz, CD_3OD) δ 7.60 (d, *J* = 15.9 Hz, 1H), 7.44 (t, *J* = 7.7 Hz, 2H), 6.80 (t, *J* = 5.7 Hz, 2H), 6.28 (d, *J* = 15.9 Hz, 1H),

3.35 (s, 1H). ^{13}C NMR (100 MHz, CD_3OD) δ 170.1, 160.1, 145.6, 130.1, 126.2, 115.8, 114.6.

*Caffeic acid (**30**)* (Nilsson et al., 2004)

^{1}H NMR (400 MHz, CD_3OD) δ 7.53 (d, *J* = 15.9 Hz, 1H), 7.04 (d, *J* = 1.9 Hz, 1H), 6.93 (dd, *J* = 8.1, 2.0 Hz, 1H), 6.78 (d, *J* = 8.2 Hz, 1H), 6.22 (d, *J* = 15.9 Hz, 1H). ^{13}C NMR (100 MHz, CD_3OD) δ 171.1, 149.4, 147.0, 146.8, 127.8, 122.8, 116.5 115.5, 115.1.

*Hesperetic acid (**31**)* (Salum et al., 2010)

^{1}H NMR (400 MHz, DMSO-*d6*) δ 7.44 (d, *J* = 15.9 Hz, 1H), 7.26 (d, *J* = 1.8 Hz, 1H), 7.05 (dd, *J* = 8.1, 6.3 Hz, 1H), 6.79 (d, *J* = 8.1 Hz, 1H), 6.37 (d, *J* = 15.9 Hz, 1H), 1.22 (s, 1H), 1.14 (d, *J* = 6.7 Hz, 3H). ^{13}C NMR (150 MHz, DMSO-*d6*) δ 149.0, 147.9, 146.8, 125.9, 124.2, 122.6, 115.5, 114.8, 114.1, 111.0, 63.1, 55.6, 55.4.

*Resveratrol (**32**)* (Huang et al., 2000)

^{1}H NMR (400 MHz, Acetone-*d6*) δ 8.45 (s, 1H), 8.19 (s, 2H), 7.42 (d, *J* = 8.6 Hz, 2H), 7.02 (d, *J* = 16.3 Hz, 1H), 6.89 (d, *J* = 16.3 Hz, 1H), 6.84 (d, *J* = 8.6 Hz, 2H), 6.55 (d, *J* = 2.1 Hz, 2H), 6.28 (t, *J* = 1.9 Hz, 1H). ^{13}C NMR (101 MHz, Acetone-*d6*) δ 159.5, 158.1, 140.9, 129.9, 129.1, 128.7, 126.8, 116.4, 105.7, 102.6.

*3,3',4,4'-Tetrahydroxybiphenyl (**33**)* (Daniela et al., 2007)

^{1}H NMR (400 MHz, CD_3OD) δ 6.84 (d, *J* = 1.8 Hz, 1H), 6.80 (d, *J* = 8.1 Hz, 1H), 6.72 (dd, *J* = 8.1, 1.9 Hz, 1H). ^{13}C NMR (100 MHz, CD_3OD) δ 146.4, 145.4, 134.9, 119.3, 116.3, 114.9.

*Phenethyl β-D-glucopyranoside (**34**)* (Zhi-Jun et al., 2013)

^{1}H NMR (400 MHz, CD_3OD) δ 7.32 (br.s, 4H), 4.37 – 4.26 (m, 1H), 4.10 (d, *J* = 7.9 Hz, 1H), 3.94 – 3.63 (m, 2H), 3.19 (m, 2H), 2.97 (dd, *J* = 15.0, 7.6 Hz, 1H), 1.03 – 0.92 (m, 1H). ^{13}C NMR (100 MHz, CD_3OD) δ 140.0, 130.0, 129.3, 127.2, 104.3, 78.1, 77.9, 75.1, 71.7, 71.6, 62.7, 37.2.

*(2S)-2-hydroxy-2-(4-hydroxyphenyl)ethyl β-D-Glucopyranoside (**35**)* (Kurimoto et al., 2011)

^{1}H NMR (400 MHz, CD_3OD) δ 7.32 (d, *J* = 8.3 Hz, 1H), 6.79 (d, *J* = 8.4 Hz, 1H), 5.91 (d, *J* = 3.9 Hz, 1H), 5.29 (d, *J* = 9.2 Hz, 1H), 3.80 – 3.65 (m, 2H), 3.51 (dd, *J* = 14.0, 9.4 Hz, 1H), 3.20 – 3.01 (m, 5H). ^{13}C NMR (100 MHz, CD_3OD) δ 157.1, 130.5, 116.3, 115.9, 104.5, 97.3, 96.3, 83.4, 77.9, 77.8, 77.5, 75.5, 71.5, 62.9, 49.6, 49.4, 49.2, 49.0, 48.8, 48.6 48.4.

*Phenethyl O-α-L-rhamnopyranosyl-(1→6)-β-D-glucopyranoside (**36**)* (Wei-dong et al., 2006)

^{1}H NMR (400 MHz, C_5D_5N) δ 7.56 (s, 1H), 7.48 (d, *J* = 4.2 Hz, 1H), 7.37 (s, 2H), 7.30 (t, *J* = 7.2 Hz, 2H), 7.27 – 7.24 (m, 1H), 6.85 (d, *J* = 5.0 Hz, 1H), 6.75 (d, *J* = 4.2 Hz, 1H), 6.65 (d, *J* = 5.9 Hz, 1H), 5.61 (s, 1H), 5.20 (d, *J* = 11.8 Hz, 1H), 4.95 (d, *J* = 7.7 Hz, 1H), 4.86 (d, *J* = 11.8 Hz, 1H), 4.73 (d, *J* = 11.2 Hz, 1H), 4.65 (d, *J* = 17.1 Hz, 1H), 4.64 – 4.53 (m, 1H), 4.44 (dq, *J* = 12.2, 6.1 Hz, 1H),

4.32 (td, *J* = 8.9, 4.8 Hz, 1H), 4.25 (t, *J* = 11.1 Hz, 2H), 4.16 – 4.02 (m, 3H). ^{13}C NMR (100 MHz, C_5D_5N) δ 139.1, 129.0, 128.9, 128.2, 104.1, 103.0, 78.8, 77.5, 75.5, 74.4, 73.1, 72.7, 72.2, 71.2, 70.2, 68.7, 18.9.

*(Z)-3-Hexen-1-ol β-D-glucopyranoside (**37**)* (Suh et al., 2002)

^{1}H NMR (400 MHz, CD_3OD) δ 5.56 – 5.32 (m, 2H), 4.30 (d, *J* = 7.8 Hz, 1H), 3.94 – 3.83 (m, 2H), 3.69 (dd, *J* = 11.9, 5.0 Hz, 1H), 3.57 (dt, *J* = 9.5, 7.2 Hz, 1H), 3.44 – 3.24 (m, 4H), 3.20 (dd, *J* = 9.0, 7.8 Hz, 1H), 2.40 (q, *J* = 7.1 Hz, 2H), 2.10 (p, *J* = 7.4 Hz, 2H), 0.99 (t, *J* = 7.6 Hz, 3H). ^{13}C NMR (100 MHz, CD_3OD) δ 134.5, 125.8, 104.3, 78.1, 77.9, 75.1, 71.6, 70.5, 62.7, 28.7, 21.5, 14.6.

*Euscaphic acid (**38**)* (Rocha Gleice da et al., 2007)

^{1}H NMR (400 MHz, CD_3OD) δ 5.30 (t, *J* = 3.3 Hz, 1H), 4.02 – 3.86 (m, 1H), 3.61 (q, *J* = 7.1 Hz, 1H), 2.59 (td, *J* = 13.2, 4.5 Hz, 1H), 2.50 (s, 1H), 2.11 – 1.92 (m, 3H), 1.89 – 1.68 (m, 5H), 1.68 – 1.37 (m, 9H), 1.35 (s, 3H), 1.34 – 1.21 (m, 6H), 1.20 (s, 3H), 1.18 – 1.15 (m, 1H), 0.99 (s, 6H), 0.93 (d, *J* = 6.7 Hz, 3H), 0.87 (s, 3H), 0.79 (s, 3H). ^{13}C NMR (100 MHz, CD_3OD) δ 182.3, 140.1, 129.4, 80.1, 73.6, 67.2, 55.1, 49.3, 48.2, 43.1, 42.7, 42.5, 41.3, 39.5, 39.4, 39.0, 34.1, 29.6, 29.2, 27.3, 27.1, 26.6, 24.9, 24.7, 22.4, 19.3, 17.5, 16.9, 16.6.

*(3β)-stigmast-5-en-3-yl-β-D-Galactopyranoside (**39**)* (Saied and Begum, 2004)

^{1}H NMR (400 MHz, DMSO-*d*6) δ 5.32 (m, 1H), 4.85 (m, 3H), 4.31 (m, 2H), 3.52-2.97 (m, glucosidic protons), 2.05-1.07 (m, 3H), 1.05 – 0.71 (m, 15H), 0.65 (s, 3H). ^{13}C NMR (100 MHz, DMSO-*d*6) δ 140.5, 121.2, 100.8, 76.9, 76.7, 73.5, 70.1, 61.1, 56.2, 55.4, 49.6, 45.2, 41.8, 38.3, 36.8, 36.2, 35.5, 33.4, 31.4, 31.4, 29.3, 28.7, 27.7, 25.5, 23.8, 22.6, 20.6, 19.7, 19.1, 18.9, 18.6, 11.8, 11.7.

S2. Structure elucidation of compound 18

(-)-(8S,7'R,8'S)-Burselignan-9'-O-a-L-rhamnoside (18)

$C_{26}H_{34}O_{10}$, amorphous solid; $[\alpha]_D^{25}$ –27.0° (c = 0.1, MeOH); ESI-MS m/z: 507.22 $[M+H]^+$; UV (MeOH) λ_{max} 203, 284 nm; ^{1}H NMR (CD_3OD, 400 MHz) and ^{13}C NMR data (CD_3OD, 100 MHz) (see Figure S1 and Table S1).

We find only one reference for (-)-(8*S*,7'*R*,8'*S*)-Burselignan-9'-*O-a-L*-rhamnoside (Zhou et al., 2016), in which, its ^{1}H, ^{13}C NMR and 2DNMR data, as well as the optical rotation and CD spectral data were reported as a novel compound. Comparing the $^{1}HNMR$ and $^{13}CNMR$ data of compound **18** with that of (-)-(8*S*,7'*R*,8'*S*)-Burselignan-9'-*O-a-L*-rhamnoside in this reference, it was found there existed a lot of differences in chemical shifts δ and coupling constant *J* . For the determination of stereochemistry of compound 18, we further obtained its specific rotation, 2D-NMR or CD spectra (see Figure S2–S8).

Then we conducted spectral analysis and compared them with the optical rotation data, CD, HMQC, HMBC, COSY, NOESY spectrum of (-)-(8*S*,7'*R*,8'*S*)-Burselignan-9'-*O-a-L*-rhamnoside reported in the reference, and found that they are the same absolute configuration. These results indicate that different solvents may have a great influence in chemical shifts and coupling constant.

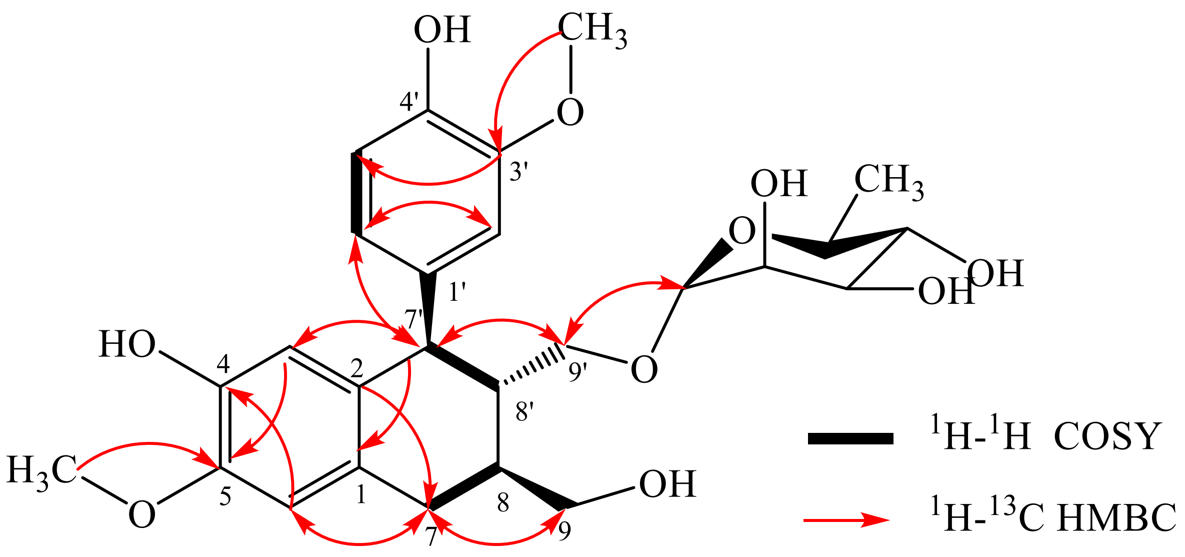

Figure S1. HMBC, COSY correlations of compound **18**

Table S1. Comparing the data ^{1}HNMR and ^{13}CNMR data of compound **18** with reference

	^{1}H-Compound **7** from Reference (500 MHz, DMSO-d6)	^{13}C-Compound **7** from Reference (125 MHz, DMSO-d6)	^{1}H- Compound **18** from TH (400 MHz, CD_3OH)	^{13}C-Compound **18** from TH (100 MHz, CD_3OH)
1		126.9		128.9
2		132.5		130.0
3	6.08 s	116.1	6.17, s	117.1
4		144.2		141.1
5		145.6		146.1
6	6.61 s	111.9	6.67, s	112.5
7	2.73, 2.72	32.3	2.83 (d, *J* = 7.4 Hz)	33.6
8	1.89, m	37.7	1.86 (t, *J* = 10.1 Hz)	45.5
9	3.55(dd, *J* = 10.0, 3.0 Hz)<br>3.41	63.1	3.74, m<br>3.68 – 3.60, m	63.4
1’		136.4		134.0
2’	6.63 br s	113.3	6.64 (d, *J* = 1.8 Hz)	113.5
3’		147.4		147.3
4’		144.8		145.2
5’	6.70 (d, *J* = 8.0 Hz)	115.4	6.76 (d, *J* = 8.0 Hz)	116.1
6’	6.59 (dd, *J* = 8.0, 1.5 Hz)	121.4	6.59 (dd, *J* = 8.0, 1.9 Hz)	123.1
7’	3.75 (d, J = 10.5 Hz)	46.5	3.87 (d, J = 10.4 Hz)	49.6
8’	1.80, m	43.4	2.03, m	40.1
9’	3.34<br>3.15	65.7	3.83, m<br>3.12 (dd, *J* = 9.8, 3.6 Hz)	65.4
1’’	4.44 br s	100.5	4.52 (d, *J* = 1.4 Hz)	102.3
2’’	3.60	70.7	3.74, m	70.1
3’’	3.49	70.9	3.52, m	70.3
4’’	3.17	72.0	3.36, m	72.5
5’’	3.48	68.6	3.52, m	68.0
6’’	1.06 (d, *J* = 6.0 Hz)	17.8	1.19 (d, *J* = 6.2 Hz)	17.9
5-OMe	3.70, s	55.5	3.81, s	56.4
3’-OMe	3.71, s	55.6	3.77, s	56.4

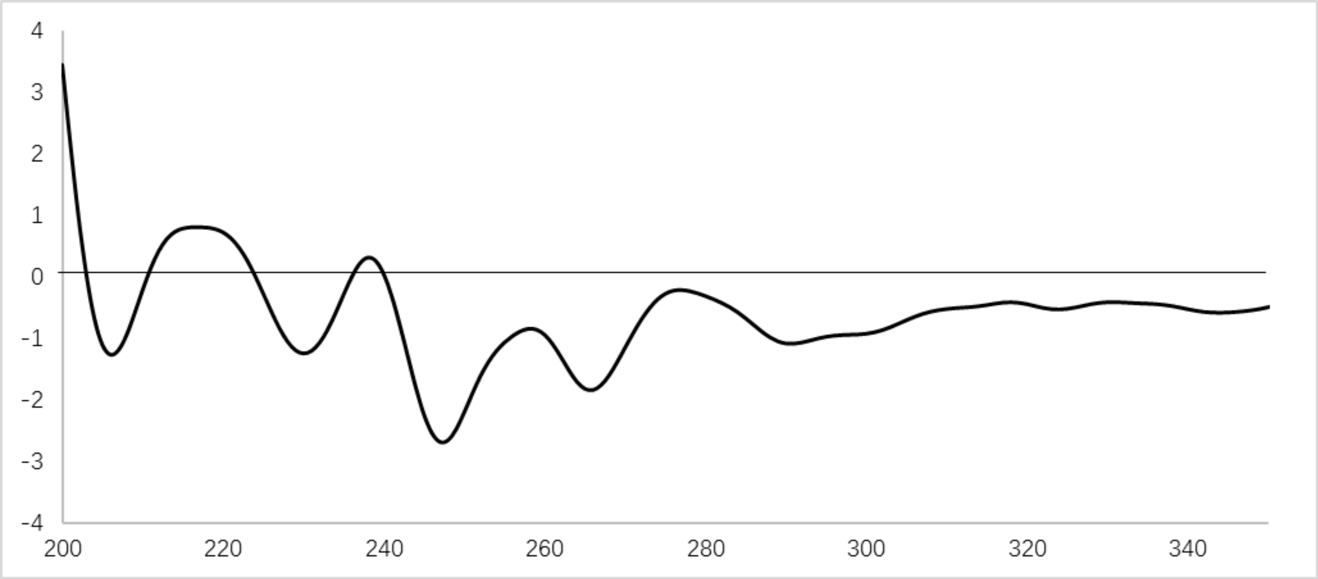

Figure S2. CD spectrum of compound **18**

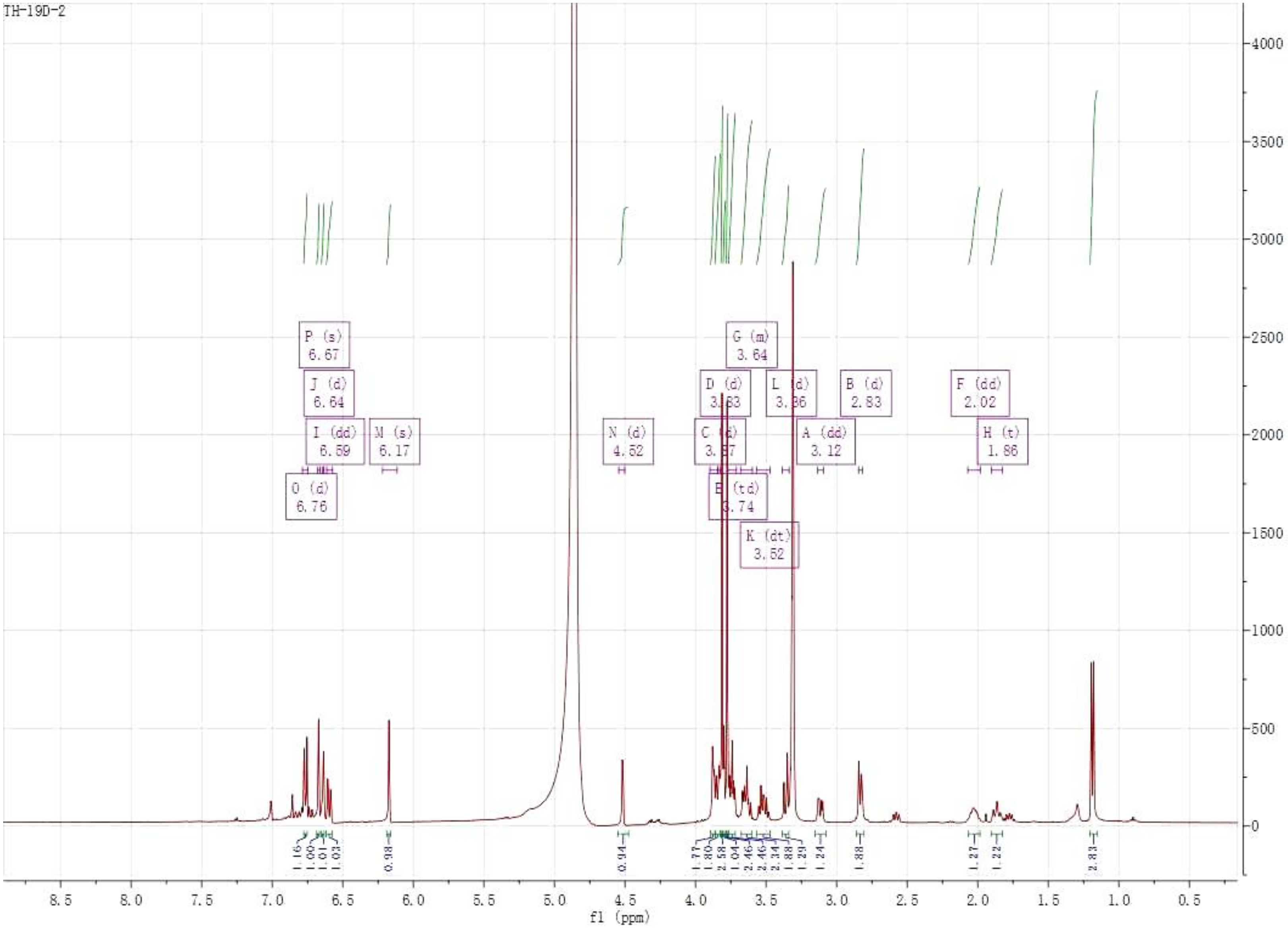

Figure S3. ^{1}H-NMR spectrum of compound **18** in CD_3OD (400 MHz)

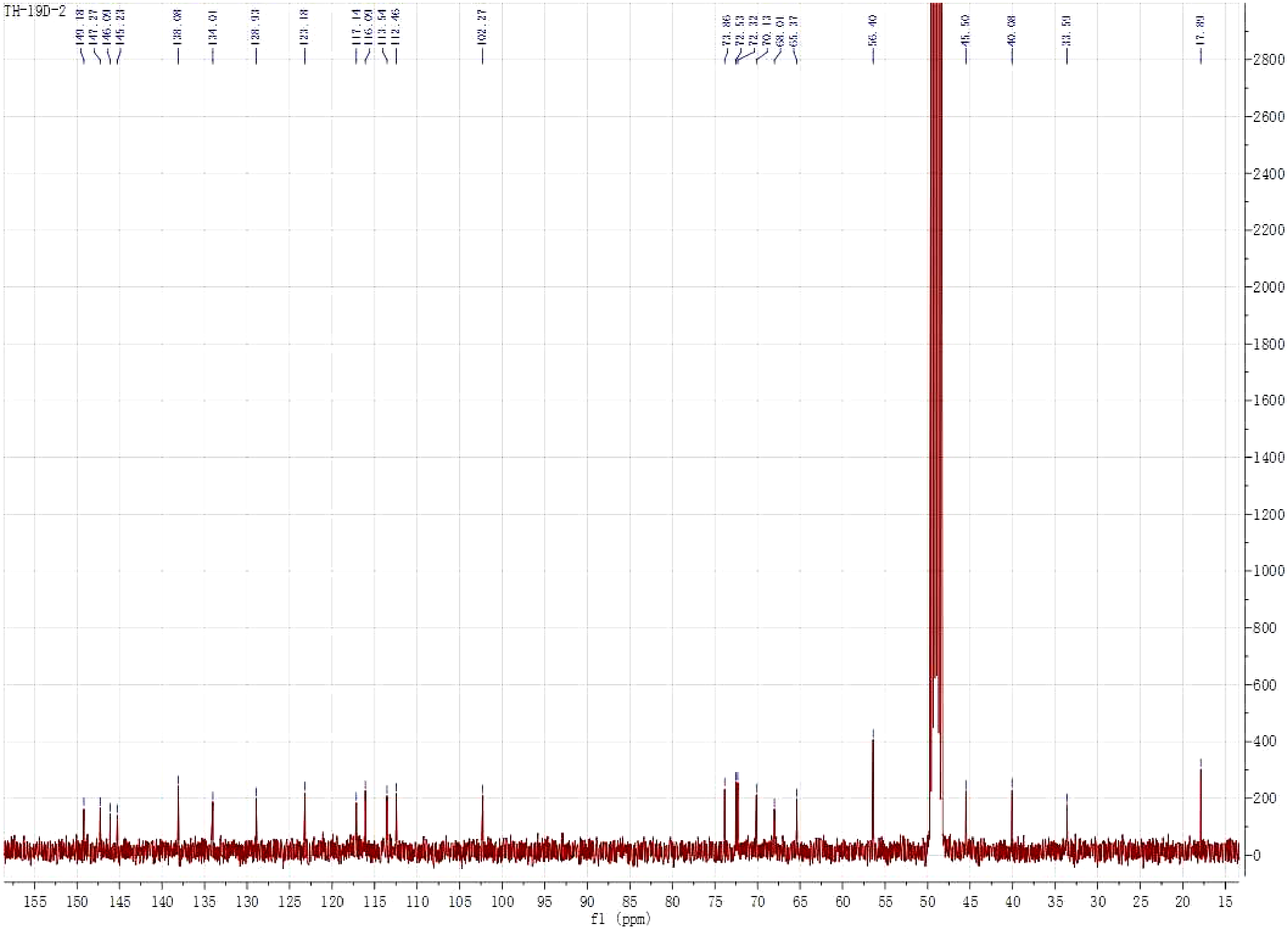

Figure S4. ^{13}C-NMR spectrum of compound **18** in CD_3OD (100 MHz)

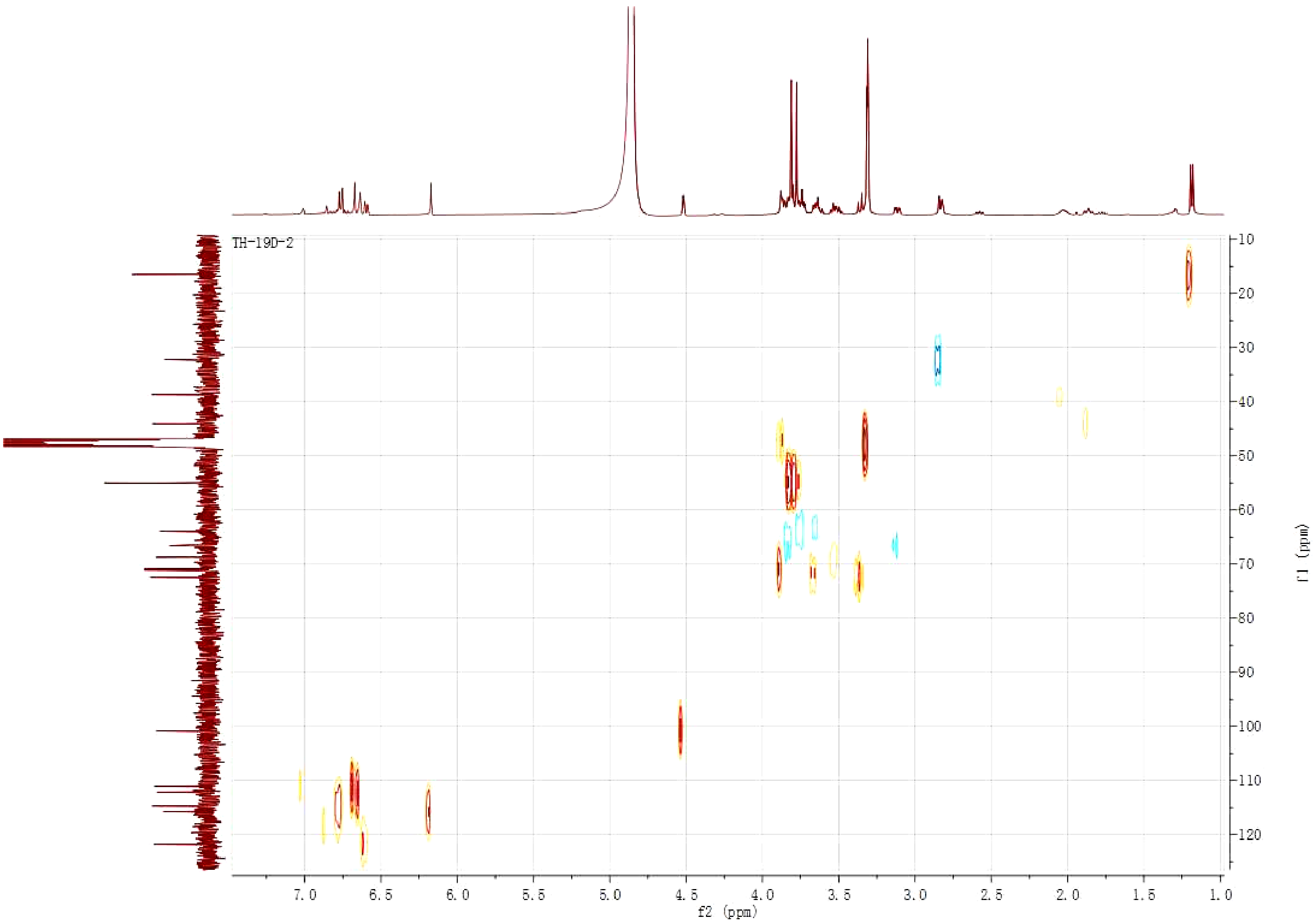

Figure S5. HMQC spectrum of compound **18** in CD_3OD (400 MHz)

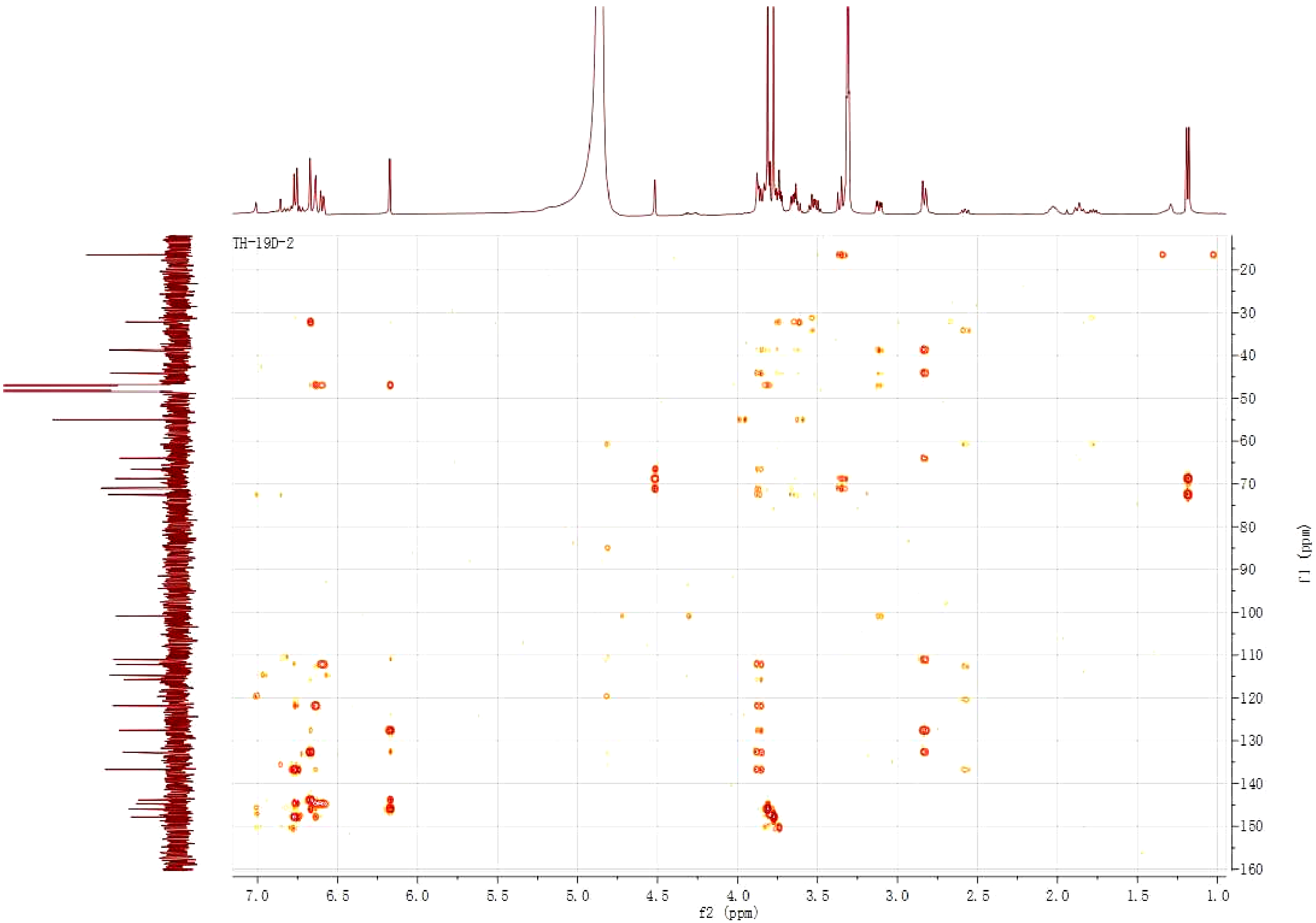

Figure S6. HMBC spectrum of compound **18** in CD_3OD (400 MHz)

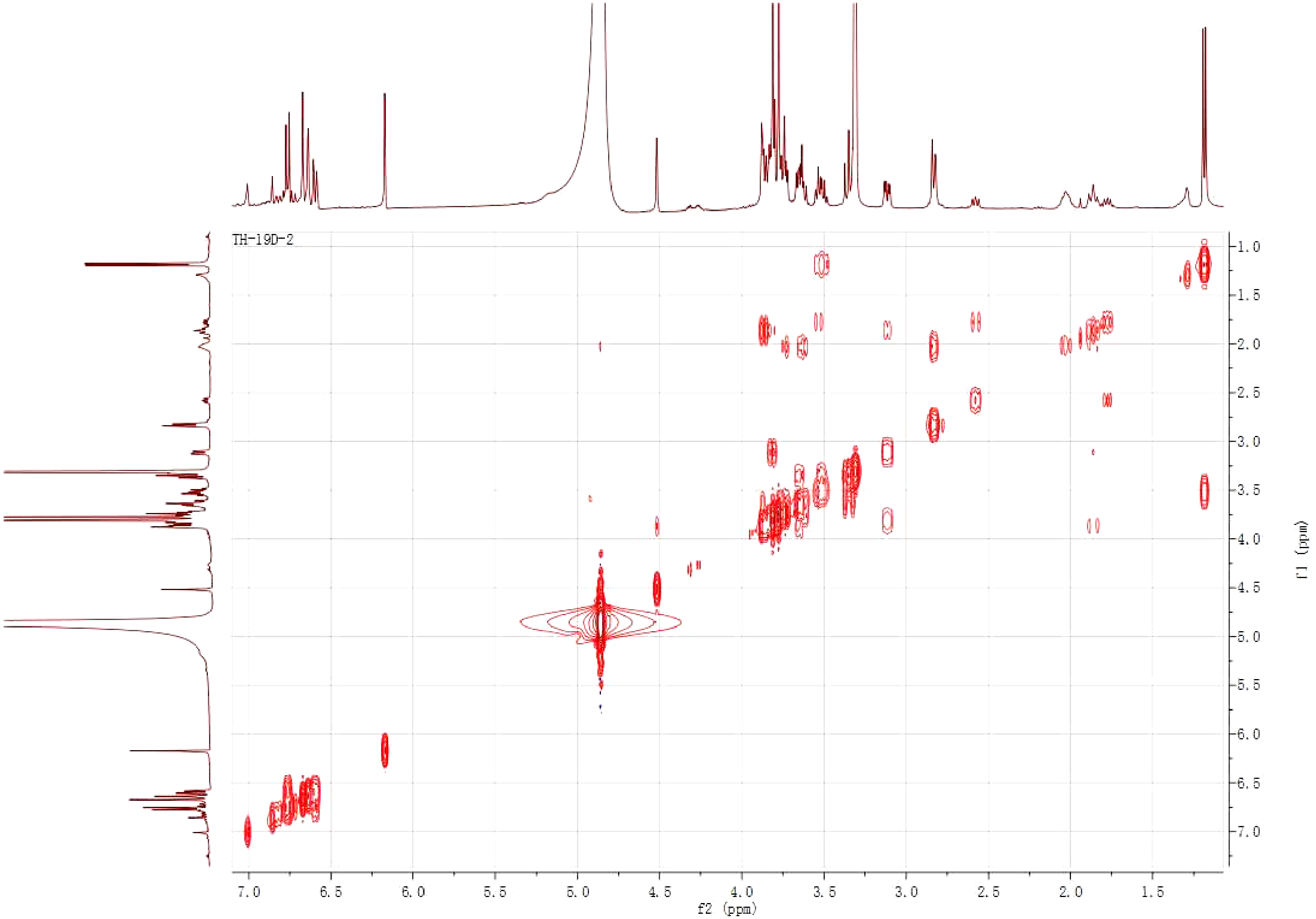

Figure S7. COSY spectrum of compound **18** in CD_3OD (400 MHz)

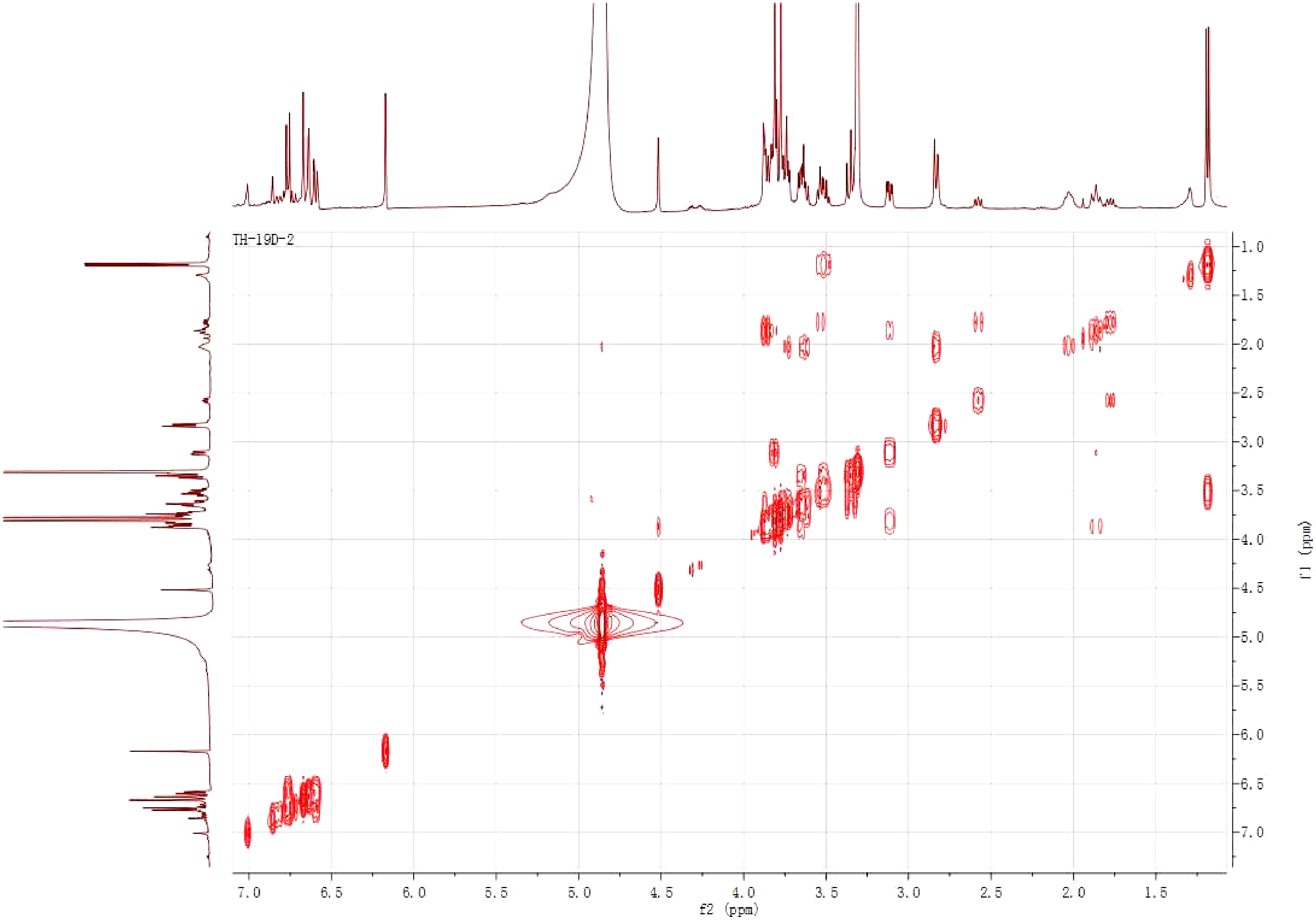

Figure S8. NOESY spectrum of compound **18** in CD_3OD (400 MHz)

S3. MTT assay of compounds

		1	2	3	Mean	Viability%
Control		2.601	3.045	2.899	2.848	139.12
LPS		2.045	2.097	2	2.047	100.00
Dex	10	2.265	2.308	2.224	2.266	110.66
TH_Ext.	50	2.085	2.128	1.799	2.004	97.88
	25	1.975	2.057	1.853	1.962	95.82
	12.5	1.958	1.783	1.833	1.858	90.75
38	50	1.972	2.166	2.093	2.077	101.45
	25	1.981	2.161	2.248	2.130	104.04
	12.5	1.932	2.091	2.176	2.066	100.93
16	50	1.937	2.207	2.068	2.071	101.14
	25	2.025	2.146	2.145	2.105	102.83
	12.5	2.067	2.198	2.145	2.137	104.36
4	50	2	2.036	2.042	2.026	98.96
	25	1.979	2.04	2.106	2.042	99.72
	12.5	1.95	2.096	1.978	2.008	98.08
19	50	2.078	2.267	2.048	2.131	92.7
	25	2.411	2.159	2.198	2.256	98.1
	12.5	2.247	2.126	2.128	2.167	94.3
5	50	2.241	2.241	2.299	2.260	98.3
	25	2.214	2.302	2.215	2.244	97.6
	12.5	2.191	2.324	2.093	2.203	95.8
6	50	2.315	2.128	2.014	2.152	93.6
	25	2.337	2.183	2.196	2.239	97.4
	12.5	2.159	2.171	1.982	2.104	91.5
8	50	2.439	2.195	2.182	2.272	98.8
	25	2.411	2.327	2.388	2.375	103.3
	12.5	2.359	2.355	2.206	2.307	100.3

MTT assay		1	2	3	Mean	Viability%
Control		0.401	0.466	0.437	0.435	100.0
LPS		0.423	0.443	0.445	0.437	100.5
32	50	0.496	0.53	0.528	0.518	119.2
	25	0.487	0.484	0.474	0.482	110.8
	5	0.466	0.433	0.436	0.445	102.4
10	50	0.417	0.406	0.377	0.400	92.0
	25	0.53	0.485	0.502	0.506	116.3
	5	0.47	0.464	0.447	0.460	105.9
18	50	0.476	0.514	0.514	0.501	115.3
	25	0.452	0.454	0.428	0.445	102.3
	5	0.43	0.425	0.395	0.417	95.9
13	50	0.43	0.423	0.399	0.417	96.0
	25	0.45	0.425	0.421	0.432	99.4
	5	0.468	0.463	0.422	0.451	103.8
12	10	0.503	0.494	0.509	0.502	115.5
	5	0.517	0.438	0.47	0.475	109.3
	1	0.423	0.408	0.447	0.426	98.0
35	10	0.485	0.403	0.446	0.445	102.3
	5	0.413	0.403	0.442	0.419	96.5
	1	0.406	0.412	0.435	0.418	96.1

S4. Interaction of molecular modeling between sEH and A/S38_601, compound 10, 12

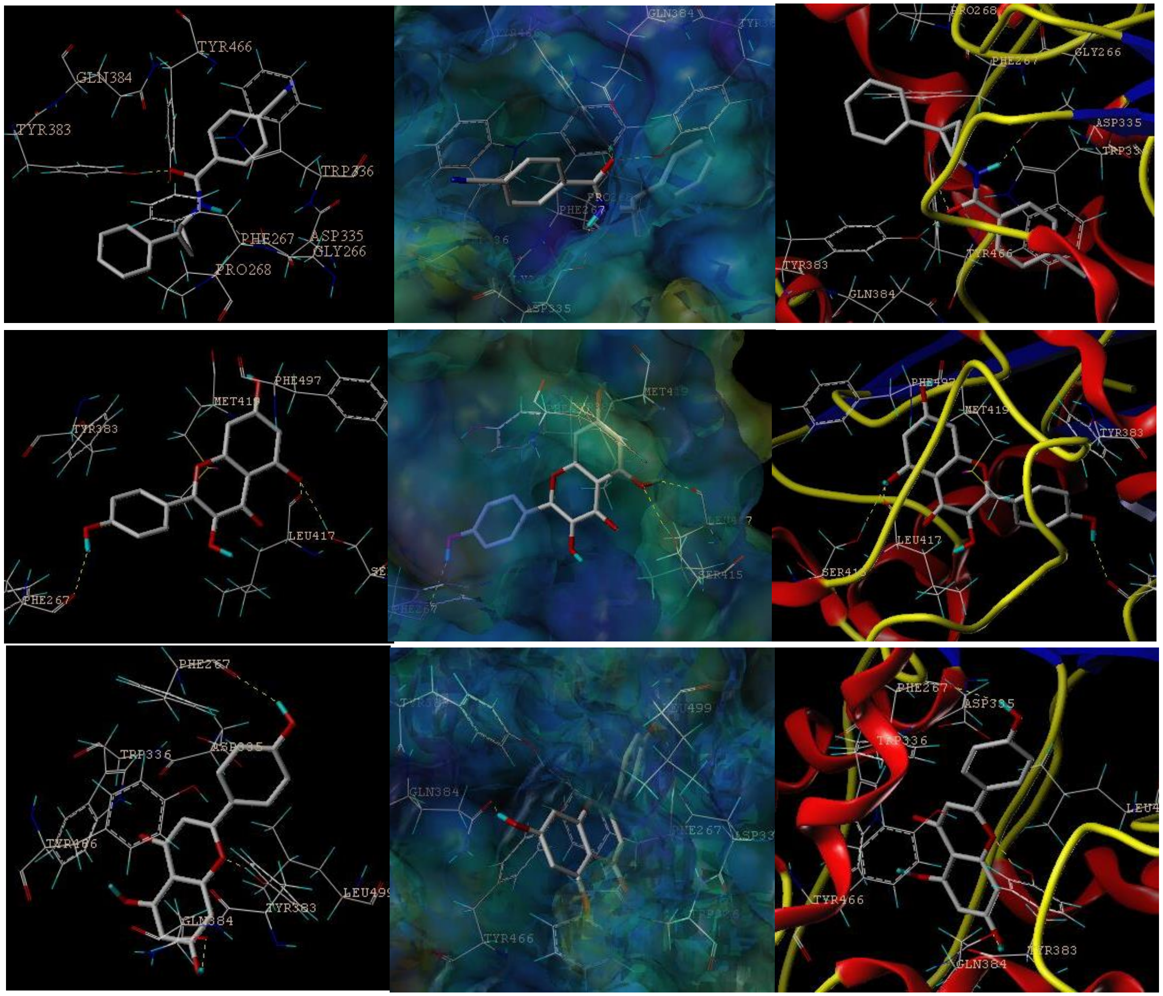

S5. Interaction of molecular modeling between iNOS and AT2_1906, compound 10, 12

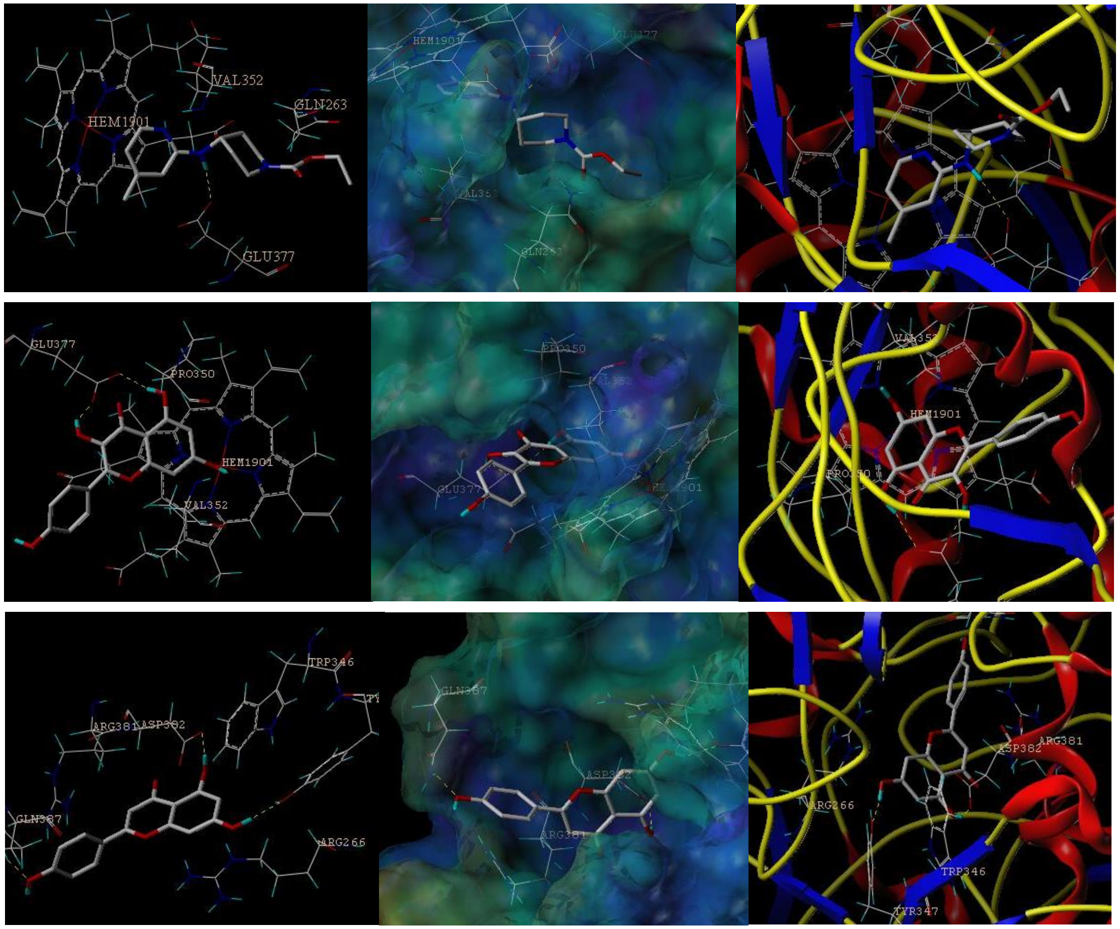

References

Bai M-M, Shi W, Tian J-M, Lei M, Kim JH, Sun YN, Kim YH, Gao J-M. Soluble epoxide hydrolase inhibitory and anti-inflammatory components from the leaves of *Eucommia ulmoides* Oliver (Duzhong). J Agric Food Chem 2015;63(8):2198-205.

Blanco, F., Alkorta, I., Elguero, J., 2007. Statistical analysis of ^{13}C and ^{15}N NMR chemical shifts from GIAO/B3LYP/6-311 + + G** calculated absolute shieldings. Magn. Reson. Chem. 45(9), 797-800.

Chae, S.-W., Lee, S.-H., Kang, S.-S., Lee, H.-J., 2002. Flavone glucosides from the leaves of helianthus tuberosus. Nat. Prod. Sci. 8(4), 141-143.

Chang, C.j., Floss, H.G., Steck, W., 1977. Carbon-13 magnetic resonance spectroscopy coumarins. Carbon-13-proton long-range couplings. J. Org. Chem. 42(8), 1337-1377.

Chang, Y.C., Chang, F.R., Wu, Y.C., 2000. The constituents of Lindera glauca. J. Chin. Chem. Soc. 47(2), 373-380.

Chin Y-W, Chai H-B, Keller WJ, Kinghorn AD. Lignans and other constituents of the fruits of *Euterpe oleracea* (Acai) with antioxidant and cytoprotective activities. J Agric Food Chem 2008;56(17):7759-64

Daniela, S., Pellegrino, C., Alessandro, P., 2007. Structural characterization of isomeric dimers from the oxidative oligomerization of catechol with a biomimetic catalyst. Biomacromolecules 8(2), 737-780.

dos Santos, A.R., de Barros, M.P., Santin, S.M.D., Sarragiotto, M.H., de Souza, M.C., Eberlin, M.N., Meurer, E.C., 2004. Polar constituents of the leaves of Machaonia brasiliensis (Rubiaceae). Quim. Nova 27(4), 525-527.

Godecke, T., Kaloga, M., Kolodziej, H., 2005. A phenol glucoside, uncommon Coumarins and flavonoids from Pelargonium sidoides DC. Zeitschrift Fur Naturforschung Section B-a Journal of Chemical Sciences 60(6), 677-682.

Huang, K.S., Wang, Y.H., Li, R.L., Lin, M., 2000. Five new stilbene dimers from the lianas of Gnetum hainanense. J. Nat. Prod. 63(1), 86-95.

Itoh, T., Ninomiya, M., Yasuda, M., Koshikawa, K., Deyashiki, Y., Nozawa, Y., Akao, Y., Koketsu, M., 2009. Inhibitory effects of flavonoids isolated from Fragaria ananassa Duch on IgE-mediated degranulation in rat basophilic leukemia RBL-2H3. Biorg. Med. Chem. 17(15), 5374-5379.

Iwai, K., Kishimoto, N., Kakino, Y., Mochida, K., Fujita, T., 2004. In vitro antioxidative effects and tyrosinase inhibitory activities of seven hydroxycinnamoyl derivatives in green coffee beans. J. Agric. Food Chem. 52(15), 4893-4898.

Kasper, J., Melzig, M.F., Jenett-Siems, K., 2010. New Phenolic Compounds of Acmella ciliata. Planta Med. 76(6), 633-U632.

Kelvin, K.O., Youla, S.T., 1991. Determination of the absolute stereochemistry of the fungal metabolite (R)-(−)-2-(4′-hydroxyphenyl)-2-hydroxyethanoic acid (pisolithin B). Can. J. Chem. 69(5), 772-778.

Kuczkowiak, U., Petereit, F., Nahrstedt, A., 2014. Hydroxycinnamic Acid Derivatives Obtained from a Commercial Crataegus Extract and from Authentic Crataegus spp. Sci. Pharm. 82(4), 835-846.

Kurimoto, S.-i., Okasaka, M., Kashiwada, Y., K, K.O., Yoshihisa, T., 2011. Four new glucosides from the aerial parts of Mediasia

macrophylla. J. Nat. Med. 65(1), 180-185.
Lai, A., Monduzzi, M., Saba, G., 1985. Carbon-13 NMR studies on catechol, phenol and benzene derivatives of biological relevance. Magn. Reson. Chem. 23(5), 379-383.
Markham, K. R., Ternai, B., Stanley, R., Geiger, H., Mabry, T. J., 1978. Carbon-13 NMR studies of flavonoids—III: Naturally occurring flavonoid glycosides and their acylated derivatives. Tetrahedron 34(9), 1389-1397.
Miyazawa, M., Oshima, T., Tokura, M., Masayoshi, H., 2003. Suppression of Chemical Mutagens-Induced SOS Response by Phenolic Acids from Black Rice Bran Using Salmonella typhimurium TA1535/pSK1002 umu Test. JOURNAL OF OLEO SCIENCE 52(9), 471-481.
Nagao, T., Abe, F., Kinjo, J., Okabe, H., 2002. Antiproliferative constituents in plants 10. Flavones from the leaves of Lantana montevidensis BRIQ. and consideration of structure-activity relationship. Biol. Pharm. Bull. 25(7), 875-879.
Nilsson, M., Duarte, I. F., Almeida, C., Delgadillo, I., Goodfellow, B. J., Gil, A. M., Morris, G. A., 2004. High-resolution NMR and diffusion-ordered spectroscopy of port wine. J. Agric. Food Chem. 52(12), 3736-3779.
Pouységu, L., Sylla, T., Garnier, T., Rojas Luis, B., Charris, J., Deffieux, D., Quideau, S., 2010. Hypervalent iodine-mediated oxygenative phenol dearomatization reactions. Tetrahedron 66(31), 5908-5917.
Rocha Gleice da, G., Simões, M., Lúcio Kelly, A., Oliveira Rodrigo, R., Coelho Kaplan Maria, A., Gattass Cerli, R., 2007. Natural triterpenoids from Cecropia lyratiloba are cytotoxic to both sensitive and multidrug resistant leukemia cell lines. Biorg. Med. Chem. 15(23), 7355-7360.
Saied, S., Begum, S., 2004. Phytochemical Studies of Berberis vulgaris. Chem. Nat. Compd. 40(2), 137-140.
Salum, M. L., Robles, C. J., Erra-Balsells, R., 2010. Photoisomerization of ionic liquid ammonium cinnamates: one-pot synthesis-isolation of Z-cinnamic acids. Org. Lett. 12(21), 4808-4819.
Sarika, S., Reddy, M.L.P., Alan, H.C., Kalyan, V.V., 2010. Synthesis and crystal structures of lanthanide 4-benzyloxy benzoates: influence of electron-withdrawing and electron-donating groups on luminescent properties. Dalton Transactions 39(3), 776-862.
Su D, Tang W, Hu Y, Liu Y, Yu S, Ma S, Qu J, Yu D. Lignan glycosides from Neoalsomitra integrifoliola. J Nat Prod 2008;71(5):784-8.
Suh, J., Jo, Y., Kim, N. D., Bae, S. J., Jung, J. H., Im, K. S., 2002. Cytotoxic constituents of the leaves ofIxeris sonchifolia. Arch. Pharmacal Res. 25(3), 289-292.
Tanaka, Y., Yanagida, A., Komeya, S., Kawana, M., Honma, D., Tagashira, M., Kanda, T., Shibusawa, Y., 2014. Comprehensive Separation and Structural Analyses of Polyphenols and Related Compounds from Bracts of Hops (Humulus lupulus L.). J. Agric. Food Chem. 62(10), 2198-2206.
Xuan, W. D., Chen, H. S., Bian, J., 2006. A new indole alkaloid glycoside from stems of Nauclea officinalis. Acta Pharm. Sin. 41(11), 1064-1071.
Xiao, Y.M., Wu, Q., Wu, W.B., Zhang, Q.Y., Lin, X.F., 2005. Controllable regioselective acylation of rutin catalyzed by enzymes in non-aqueous solvents. Biotechnol. Lett. 27(20), 1591-1595.
Zhi-Jun, W., Yun-Heng, S., Wei-Dong, Z., 2013. Chemical constituents of Ainsliaea macrocephala. Chem. Nat. Compd. 49(1), 167-

169.
Zhou, J., Li, C.J., Yang, J.Z., Ma, J., Wu, L.Q., Wang, W.J., Zhang, D.M., 2016. Phenylpropanoid and lignan glycosides from the aerial parts of Lespedeza cuneata. Phytochemistry 121, 58-64.
Zhu, X., Dong, X., Wang, Y., Ju, P., Luo, S., 2005. Phenolic Compounds from Viburnum cylindricum. Helv. Chim. Acta 88(2), 339-342.
Zhu, Y., Zhang, L.X., Zhao, Y., Huang, G.D., 2010. Unusual sesquiterpene lactones with a new carbon skeleton and new acetylenes from Ajania przewalskii. Food Chem. 118(2), 228-238.
